# Genetic Mechanisms Underlying Cortical Evolution in Mammals

**DOI:** 10.3389/fcell.2021.591017

**Published:** 2021-02-15

**Authors:** Lucía Florencia Franchini

**Affiliations:** Instituto de Investigaciones en Ingeniería Genética y Biología Molecular (INGEBI), Consejo Nacional de Investigaciones Científicas y Técnicas (CONICET), Buenos Aires, Argentina

**Keywords:** brain, elephant, cetacea, primates, human, cortex, human accelerated region, synapsids

## Abstract

The remarkable sensory, motor, and cognitive abilities of mammals mainly depend on the neocortex. Thus, the emergence of the six-layered neocortex in reptilian ancestors of mammals constitutes a fundamental evolutionary landmark. The mammalian cortex is a columnar epithelium of densely packed cells organized in layers where neurons are generated mainly in the subventricular zone in successive waves throughout development. Newborn cells move away from their site of neurogenesis through radial or tangential migration to reach their specific destination closer to the pial surface of the same or different cortical area. Interestingly, the genetic programs underlying neocortical development diversified in different mammalian lineages. In this work, I will review several recent studies that characterized how distinct transcriptional programs relate to the development and functional organization of the neocortex across diverse mammalian lineages. In some primates such as the anthropoids, the neocortex became extremely large, especially in humans where it comprises around 80% of the brain. It has been hypothesized that the massive expansion of the cortical surface and elaboration of its connections in the human lineage, has enabled our unique cognitive capacities including abstract thinking, long-term planning, verbal language and elaborated tool making capabilities. I will also analyze the lineage-specific genetic changes that could have led to the modification of key neurodevelopmental events, including regulation of cell number, neuronal migration, and differentiation into specific phenotypes, in order to shed light on the evolutionary mechanisms underlying the diversity of mammalian brains including the human brain.

## Introduction and Road Map for This Review

In this review I propose a journey through the evolutionary history of the cortex in mammals. From the appearance of the six-layered neocortex in an ancestor of mammals to the evolution of the human brain. Although in this work, I compare the neocortex of mammals to homologous brain regions of other amniotes, an exhaustive comparison of the different brain plans in reptiles, birds and mammals and the different hypotheses that have been delineated to explain their evolutionary history are outside the scope of this review. For this matter excellent reviews and books are available (Northcutt and Kaas, 1995; Aboitiz et al., 2002; Striedter, 2005; Medina, 2007; Bruce, 2010; Montiel and Aboitiz, 2015; Montiel et al., 2016; Goffinet, 2017; Nomura and Hirata, 2017; Kaas, 2020). I mainly focus this review on the developmental pathways that were probably modified to render the mammalian neocortex. In addition, I analyze current knowledge about the evolution of the brain in mammalian lineages that are characterized by highly elaborated cognitive capacities such as elephants, primates and cetaceans. Finally, I concentrate on recent findings in human-specific genetic modifications and their potential impact in the evolution of the human brain.

## The Mammalian Brain

### Basic Plan

**Figure 1:**
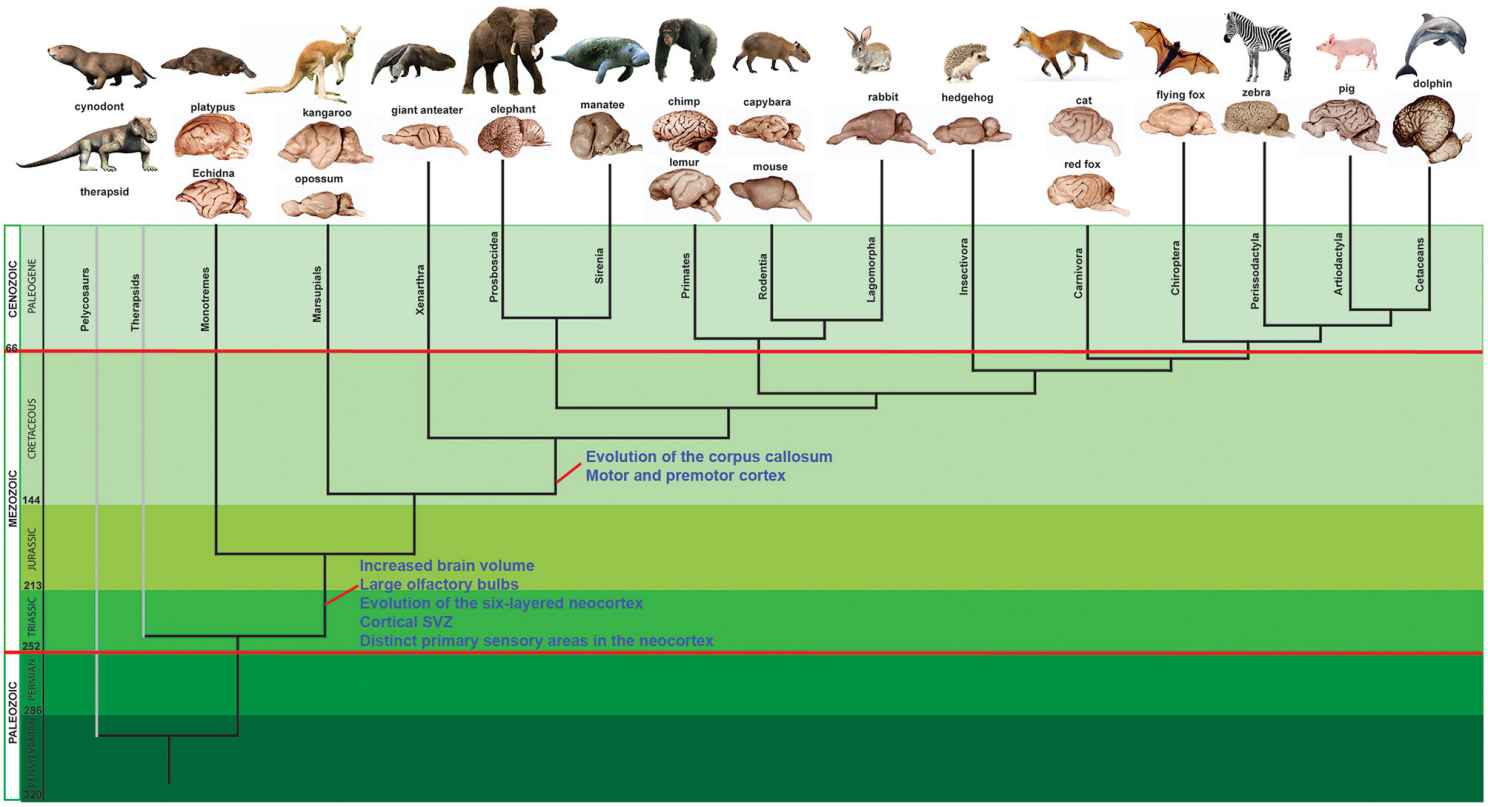
Phylogenetic tree of mammalian evolution. The schematic phylogenetic tree has been based on phylogenetic trees built by Goffinet (2017) and Rowe (2017). Red lines mark the mass extinction events. In every lineage two examples of lissencephalic and gyrencephalic brains are shown. Extinct lineages show examples of species that have been described from fossils specimens. Drawings of Therapsid Proburnetia viatkensis Tatarinov species and Cynodont Kayentatherium wellesi Kermack species were performed by the artist Nobu Tamura (http://spinops.blogspot.com/) and reproduced with permission.

Mammals are the most widespread group of vertebrates having conquered a large variety of ecological niches on land, water, and air. There are around 5,500 mammalian species today classified in 18 orders. Three subgroups of mammals are clearly distinguished among living mammals. Monotremata (Prototheria), is a group of egg-laying mammals that live in Australasia and represented today by only two species of echidna and a species of platypus (Figure 1). Marsupialia (Metatheria) are pouched mammals living today in the Americas and Australasia and classified in 260 species, the most representative of which are the kangaroos and the opossums. Placentalia (Eutheria) is the largest group, with around 4,300 species divided in 18 orders that have been clustered in four major branches: Xenarthra, encompassing anteaters, armadillos, and sloths; Afrotheria, a group including elephants and tenrecs, Laurasiatheria, with bats, cats, cows and whales; and Euarchontoglires, a group composed of rodents, primates, flying lemurs and rabbits (Figures 1, **3**).

Beyond the very well-known characteristics that distinguish mammals from other vertebrates such as hair, breast-feeding, jaws, dentition, etc., the mammalian brain allows this successful group to sense the world in a unique way. In fact, Mammals have evolved a series of innovations regarding the way they can read sensory clues, including a highly developed sense of smell and the ability to better detect and discriminate airborne sounds. On the other hand it has been hypothesized that mammals at some point became nocturnal and as a consequence they lost their ability to see color (Walls, 1942; Land and Osorio, 2003). Thus, these changes in the sensory system have also impacted in the brain centers that process sensory information. Beyond the diversity and specialization of the mammalian brain in different lineages a basic organization of the mammalian brain is characterized by a well-developed forebrain that contains a six-layered neocortex located dorsally. In fact, at the beginning of development, shortly after its closure, the neural tube forms rostrally three primary vesicles namely prosencephalon (forebrain), mesencephalon (midbrain), and rhombencephalon (hindbrain). These primary vesicles later develop into five secondary brain vesicles: whereas mesencephalon stays undivided, the prosencephalon splits to render the telencephalon and diencephalon, and the rhombencephalon is subdivided into the metencephalon and myelencephalon. From the telencephalon are developed the cerebral cortex together with several subcortical structures, including the hippocampus, basal ganglia, limbic system and the olfactory bulbs. Whereas the cerebral cortex primarily derives from the dorsal part of the telencephalon, the ventral telencephalon is composed of the ganglionic eminences (GE) from where interneurons that express the inhibitory neurotransmitter GABA originate and later migrate to the developing cortex (Gelman and Marín, 2010; Faux et al., 2012).

The cerebral cortex can be subdivided either into: isocortex and allocortex based on histological criteria; homogenetic and heterogenetic based on layer development timelines; or neocortex, paleocortex and archicortex based on evolutionary criteria. The archicortex consists of the hippocampal formation, which is located ventromedially related to the neocortex. This part of the cortex is involved in learning and memory. The paleocortex consists of the olfactory bulbs, limbic structures (amygdala), piriform cortex and secondary olfactory cortex and it is located ventrolaterally in relation to the neocortex.

The isocortex or neocortex in mammals is located dorsally and comprises the phylogenetically youngest cortical areas and it is characterized by a six-layered structure that develops during fetal stages and maintains this lamination pattern in adulthood. The neocortex mainly deals with sensory information beyond olfactory input that is processed at the piriform cortex. The neocortex is organized in regions specialized for different functions: these areas include primary visual (V1), somatosensory (S1), and auditory areas (A1). In addition there are other areas in the neocortex such as motor areas, secondary somatosensory, visual and other areas that vary from lineage to lineage.

Information from fossils (endocasts) and extant mammals is used to describe the basic brain of early mammals and protomammals. The fossil evidence indicates that early mammals had little neocortex relative to brain size and that piriform cortex and other areas dedicated to olfaction were more developed. Thus, the olfactory bulbs were quite large since early mammals had a very well-developed sense of smell. Regarding other areas of the brain, it is very probable that ancestral mammals lacked a corpus callosum that connects both cerebral hemispheres since although this structure is present in all placental mammals it is not found in monotremes or marsupials (Aboitiz and Montiel, 2003; Mihrshahi, 2006; Kaas, 2013). On the other hand, in the basal ganglia, the striatum is present in all tetrapods and receives dopaminergic projections from the diencephalum and/or the tegmentum, thus we suppose that basal ganglia were present in ancestral mammals. Moreover, other structures such as the nucleus accumbens, pallidum (globus pallidus) were also present as in all tetrapods.

### The Emergence of the Mammalian Brain: Comparison to Other Tetrapods Brains

**Figure 2:**
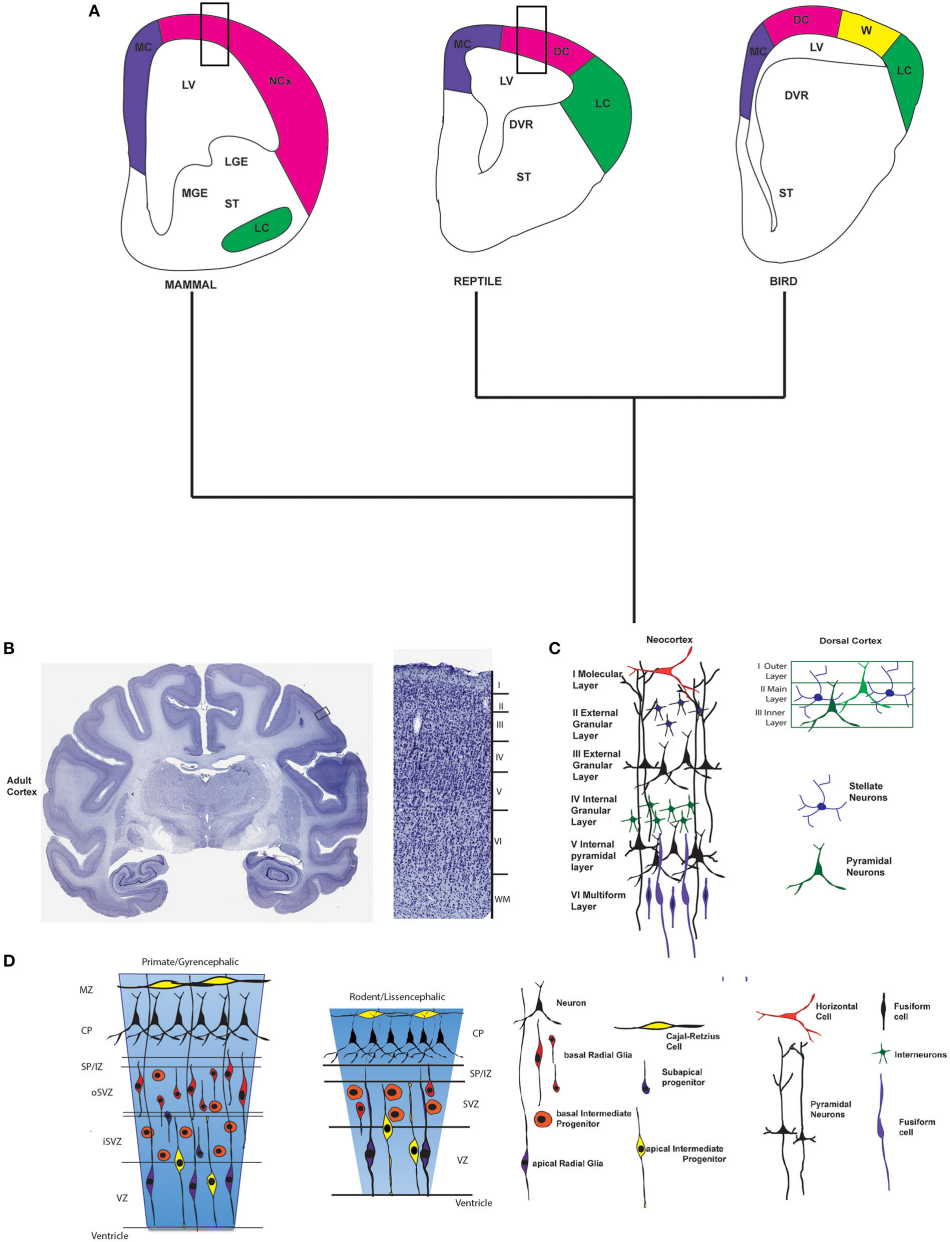
Cortex across amniota. **(A)** Schematics of coronal sections at the forebrain in amniotes. On the left a drawing of the developing mammalian forebrain (based on the mouse) indicating the location of the neocortex (NCx), medial cortex (MC), lateral cortex (LC), and ventral telencephalic structures such as the lateral and medial ganglionic eminences (LGE and MGE). In the middle and at the right schematics of the reptile and bird forebrains showing dorsal cortex (DC), medial cortex, lateral cortex, hyperpallium or Wulst (W), and subpallial structures as the dorsal ventricular ridge (DVR). The approximate location of the striatum is also indicated (ST). Colors indicate brain regions that are homologous among the different vertebrate lineages. Rectangles in mammal and reptile brains indicate approximate location of the layers schematic shown in **(C)**. **(B)** A Nissl stained coronal section of the adult macaca rhesus forebrain is shown. The rectangle indicates the approximate location of the magnification shown at the right. Magnification shows layers of the neocortex. **(C)** Schematic of the six layers of the neocortex in the adult mammalian neocortex. Next, a drawing shows the three layers of the dorsal cortex in a reptile. **(D)** Representational drawings of the developing neocortex of a gyrencephalic primate and a lissencephalic rodent where the germinative zones and cellular types are indicated. Next to it, the different cellular types of the adult and the embryonic developing neocortex are indicated. Macaque rhesus (*Macaca mulatta*) brain slices are from BrainMaps: An Interactive Multiresolution Brain Atlas; http://brainmaps.org.

What is different about the mammalian cortex compared to other tetrapods? In the reptiles the homologous forebrain region to the neocortex is the dorsal cortex but it possesses three layers of which only one possesses the neuronal bodies of pyramidal neurons and interneurons (Figure 2) (Aboitiz et al., 2002; Bruce, 2010; Molnár, 2011). In addition, reptiles and birds (sauropsids) possess a big structure in the telencephalon called the dorsal ventricular ridge (DVR) where many sensory inputs like visual, somatosensory and auditory, are processed and in this ways covers many of the functions of the mammalian neocortex (Figure 2). Several hypotheses have been proposed to explain the origin of the DVR of birds and reptiles but they are outside the reach of this review (see Striedter, 2005; Medina, 2007; Butler et al., 2011; Montiel et al., 2016; Puelles et al., 2017). In birds, although they have a large dorsal cortex, it is organized in nuclei and not in layers (Dugas-Ford et al., 2012). The dorsal cortex is called “Wulst” or hyperpallium (Reiner et al., 2004). There is almost no doubt that the Wulst is the homologous region to the dorsal cortex in reptiles and also to neocortex in mammals. However, it is small in the majority of birds compared to the mammalian neocortex and it has been suggested that it is the very big DVR in birds that plays many of the functions of the cortex in mammals (Figure 2). Since the Wulst process mainly visual and some somatosensorial inputs, it is more developed in those birds that have improved visual capacities (Striedter, 2005).

It is proposed that the stem amniotes from which mammals and present day reptiles and birds originated had a cerebral cortex in the telencephalon. In fact, a basic plan for the organization of this amniote cortex has been proposed (Puelles et al., 2016, 2017): this cortex is divided in a ventral part and three dorsal fields that includes medial, lateral, and dorsal components. Whereas, the medial part in sauropsids corresponds in mammals to the hippocampal formation, the lateral cortex coincides with the piriform cortex and the dorsal cortex corresponds to the neocortex (Puelles et al., 2016, 2017).

### How the Neocortex Is Made in Mammals?

Before analyzing the genetic pathways that could underlie the evolution of the six-layered neocortex, I will summarize briefly how the cortex develops in mammals compared to sauropsids and birds. In mammals the cortex is composed approximately of 80% of excitatory glutamatergic neurons that are generated *in situ* through the proliferation and migration of progenitor cells. In addition, the cortex possesses GABAergic cortical interneurons that originate in the ganglionic eminences and that migrate to the cortex (Gelman and Marín, 2010; Faux et al., 2012). The neocortex develops through a process called neurogenesis from a single layer of neural progenitor cells (NPCs) that cover the lateral ventricles and that are present in early stages of brain development as neuroepithelial cells (NECs). This layer of progenitor cells that covers the lateral ventricles is known as ventricular zone (VZ) (Figure 2). In early stages of development NEC divide symmetrically to amplify the progenitor pool and then, at the onset of neurogenesis NECs acquire glia markers and are from this stage named as apical radial glia cells (aRG). Then, aRG can divide symmetrically or asymmetrically to give origin either to more aRG or to three other cell types: (i) basal radial glia (bRG), (ii) intermediate progenitors (IPs), or (iii) neurons (for a review of cell types see Florio and Huttner, 2014; Goffinet, 2017) (Figure 2).

IPs migrate into a new layer or proliferative zone called the Subventricular Zone (SVZ). In the SVZ, IPs divide symmetrically to generate more IPs, before differentiating into neurons. Early born neurons, in turn migrate through the intermediate zone (IZ) to form first the preplate and later the cortical plate (CP). Neurons are organized in the CP forming layers that are deposited during development in an inside to outside manner in which layers VI and V are formed first and then IV, III and II (for a review see Rakic, 2009). Layer I, that consist mainly of Cajal-Retzius neurons, is an exception to this inside-outside pattern since these cortical cells are born earlier (around mouse embryonic days 10–11.5) and migrate to form this layer (Germain et al., 2010). Layer I is called the molecular layer and contains very few neurons and together with layer II or external granular layer, and layer III which is the external pyramidal layer constitute the supragranular layers. The supragranular layers are the primary origin and termination of intracortical connections that permits communication between one portion of the cortex and other regions (Swenson, 2006). Layer IV or internal granular layer receives thalamocortical connections, mainly from specific thalamic nuclei. Layer V called the internal pyramidal layer and layer VI known as the multiform/fusiform layer constitute the infragranular layers, which function is to connect the cerebral cortex with subcortical regions. Each cortical layer contains different cell types, for instance the pyramidal cells are the main neuronal type within layers III and V (Figure 2).

In reptiles, like the turtles, it has been described that they possess a VZ where cell division occurs, but not SVZ has been found (Cheung et al., 2007). In diapsids, like the gecko, it has been shown that NE cells divide first symmetrically and then asymmetrically to generate neurons (Nomura et al., 2013a). In addition, neurogenesis in the cortex of turtles and lizards obeys an outside-to-inside gradient (Goffinet et al., 1986). In birds (particularly in the chick), it has been shown that they have a clearly distinguished SVZ where cell divisions occur at E8 and E10. This SVZ is present in pallial and subpallial structures like the DVR and basal ganglia but not in the dorsal cortex (Cheung et al., 2007).

### Evolution of the Six-Layered Neocortex in Mammals: When, How, and Where?

To clearly establish when the first animal to be called mammal appeared on Earth depends on the definition of mammals. Mammals possess many distinctive characters but in the fossil record it is possible to find many animals that show a few but not all the characters that define mammals. The history of mammals is a very rich one and it starts very early on with the appearance of a lineage of reptiles that showed some of the distinctive mammalian characters. Here I will revise this story very briefly but excellent reviews and books on the matter can be found (Kemp, 2005; Kielan-Jaworowska et al., 2005; Rowe, 2017).

Early reptiles, now usually called “stem amniotes,” originated from amphibians about 320 million years ago in the late Carboniferous (Colbert et al., 2001; Benton, 2015; Benton et al., 2015) and soon (around 305 mya) divided into two major clades, the sauropsid or diapsid clade and the synapsid clade. From the sauropsid clade originated modern reptiles and birds, while the synapsid clade, led to the appearance of early mammals ~280 mya (Figure 1). Stem synapsids are conformed by two groups: pelycosaurs and therapsids (Figure 1). It is known that after the Permian-Triassic mass extinction 80% of terrestrial vertebrates disappeared but some therapsids survived, particularly the dicynodonts and the cynodonts (Kemp, 2005) and from this last group it is documented that the stem mammals evolved ~240 mya (Figure 1).

Thus, during the first part of the Mesozoic era the first animals that are named mammals appeared. These early mammals (or Mammaliaformes) were very small, shrew-like insectivores that were mostly nocturnal or lived underground. As mentioned before, these habits did not require three color vision, which led to the loss of opsins at some point during the evolution of mammals whereas trichromatic color vision was conserved in diapsids (Rowe et al., 2011). From this group, the egg-laying prototherians splitted very early on around 200 mya, whereas the metatherians or marsupials diverged more recently, around 150 mya from the lineage leading to Eutherian or placental mammals (Figure 1). For many years, until around 66 mya, mammals were small animals like mice, rats or shrews and some of them a little larger like cats or dogs. When dinosaurs started to disappear, around 66 mya, mammals rapidly diverged and occupied a diversity of ecological niches (Figure 1). This adaptive radiation led to the appearance of a great diversity of mammals from all the mammalian orders, some of which inhabit the Earth today.

Regarding the appearance of the six layered neocortex it is known that all therian mammals, including placentals and marsupials possess a six layered neocortex. In fact, it has been shown that marsupials display an organized SVZ, determined by the presence of basal progenitor cells and a pattern of expression of genes that resembles the one found in eutherian mammals, implying that the SVZ emerged prior to the Eutherian-Metatherian divergence (Cheung et al., 2010).

In addition, it is now known that monotremes that splitted from the mammalian lineage very early on (around 200 mya; Figure 1) after the appearance of what are called stem mammals, have a six-layered neocortex (Krubitzer et al., 1995) and also the presence of a SVZ has been described (Ashwell and Hardman, 2012). This indicated that a six-layered neocortex was already present before the split between monotremes and therian mammalian lineages. Then, the question is: did synapsids have six-layered neocortex? Undoubtedly, to answer this question we have to analyze only fossil evidence. From reconstructions performed using brain endocasts and braincases it looks like there was no great development of the telencephalon (Kemp, 2005), thus the answer to the above question is probably not. However, very recently Laaß and Kaestner have reported what seems to be the earliest evidence of a structure analogous to the mammalian neocortex in the fossorial anomodont (Therapsid) *Kawingasaurus fossilis* from the late Permian of Tanzania (Laaß and Kaestner, 2017). This finding is striking because in all therapsids the telencephalon is apparently quite narrow and does not show any clear signs of enlargement (Hopson, 2001; Kielan-Jaworowska et al., 2005; Kemp, 2009; Rowe et al., 2011). However, the authors of this finding concluded that the appearance of this neocortex-like structure is the result of convergent evolution (Laaß and Kaestner, 2017).

Thus, although this cannot be certainly established the appearance of a six-layered neocortex should have happened between the emergence of stem-mammals from therapsids (around 250 mya) and the evolution of monotremes (around 200 mya) (Figure 1).

In addition, regarding cynodonts there is a lot of discussion among specialist about the evolution of the brain in this group but the general agreement is that although it was very small compared to mammals there was some tendency to an increased size (Kemp and Parrington, 1979; Quiroga, 1980; Kemp, 2005; Kielan-Jaworowska et al., 2005).

Regarding Mammaliaformes, in addition to the general shape of the endocast that suggest an enlarged telecenplalon (Kemp and Parrington, 1979; Quiroga, 1980; Kermack and Kermack, 1984; Kielan-Jaworowska, 1986) and also the presence of a neocortex (Allman, 1999; Kielan-Jaworowska et al., 2005) there is also indirect evidence that the emergence of Mesozoic mammals marks the origin of the neocortex (Rowe, 2017). In fact, it has been suggested that the presence of a special kind of hair follicles called guard hairs involved in mechanoreception found in fossils from China (Ji et al., 2006) indicates the presence of somatosensory regions in the neocortex (Rowe, 2017).

Thus, it is apparent from the evidence analyzed so far that the expansion from a three- to a six-layered neocortex took place at some point in a Mammaliaformes in the lineage leading to the emergence of the common ancestor of all present day mammals. The emergence of a six-layered neocortex required the evolution of a developmental mechanism leading to increase neural production during embryonic neurogenesis. As mentioned before, in the mammalian embryonic cortex aRGs are the main type of progenitor cells, they form in the ventricular zone where they undergo mitosis to generate daughter cells that can take two different pathways: to leave the cell cycle and differentiate as neurons in a mechanisms known as direct neurogenesis or remain as progenitors an re-enter the cell cycle. In fact, aRGs give rise to two types of basal progenitors that migrate to build the subventricular zone (SVZ): bRGs and bIPs. These basal progenitors in turn divide to generate neurons in a two-step process known as indirect neurogenesis (Figure 2). Direct neurogenesis produces neurons in a fast way but also exhausts the progenitor pool rapidly. This is the mechanism that mainly produces neurons in the dorsal cortex of reptiles and birds. These diapsid derived vertebrates do not possess a SVZ in the homolog region of the neocortex, where indirect neurogenesis occurs in mammals (see above). Thus, it is possible that the evolution of this two-step mechanism of neurogenesis or indirect neurogenesis could be the key step in the evolution of the six-layered neocortex.

Moreover, this two-step neurogenesis mechanism that occurs in the SVZ could underlie the amplification of the number of neurons produced by increasing the pace and by lengthening the period of neurogenesis that is the raw material for the expansion of the cerebral cortex in diverse mammalian lineages.

### Cortical Folding in Mammals

The size of the neocortex varies remarkably among mammalian species. The extension of the surface area of the neocortex, results in a pattern of folds that characterizes many mammals. For excellent comprehensive reviews on the matter see (Albert and Huttner, 2015; Striedter et al., 2015; Borrell, 2018; Kroenke and Bayly, 2018; Llinares-Benadero and Borrell, 2019). Cortical folding is the result of developmental mechanisms that lead to an extension increase of cortical layers which outcome is a pattern of gyri and sulci. Cortical folding has been described only in mammals. Species without cortical folding are called lissencephalic and species displaying folded brains are named gyrencephalic. Gyrification correlates with neocortical enlargement (Reillo and Borrell, 2012; Lewitus et al., 2013) and it is not the result of a particular evolutionary trend in some mammalian groups, as it is present in all mammalian orders (Figure 1). It has been postulated that folding appeared as an evolutionary solution to the problem of increasing cortical surface area without increasing the volume of the crania (Zilles et al., 2013). However, this hypothesis has been challenged by studies focusing on developmental mechanisms (Borrell, 2018). Cortical folding has been associated with the splitting of the SVZ and the appearance of the outer SVZ (oSVZ) in several gyrencephalic species (Reillo et al., 2011). In fact, the seminal finding by Smart et al. (2002) that in rhesus monkeys the SVZ was splited into two distinctive proliferative layers, i.e., oSVZ and inner SVZ (iSVZ) led to the identification of the oSVZ, as the principal source of cortical neurons in primates (Dehay et al., 2015). The oSVZ in rhesus monkeys and humans is populated by a particular kind of progenitor cell that is collectively known as basal Radial Glia (bRGCs). These progenitors were first described in the developing human neocortex (Fietz et al., 2010; Hansen et al., 2010) and then in other gyrencephalic mammals, such as ferret, cat and sheep (Reillo et al., 2011). In contrast, in the lissencephalic mouse, the SVZ is undifferentiated and a few bRGCs have been found (Wang et al., 2011). Thus, cortical folding has been also linked to a higher abundance of bRGCs in gyrencephalic vs. lissencephalic species (Wang et al., 2011; Pilz et al., 2013). Moreover, increasing the number of bRGCs in the mouse embryonic cortex through genetic manipulations leads to the appearance of folds (Stahl et al., 2013; Florio et al., 2015; Ju et al., 2016; Wang et al., 2016). Although, some lissencephalic mammals such as the marmoset and rats display a small oSVZ (Kelava et al., 2012; Martínez-Cerdeño et al., 2012). The presence of oSVZ-like structures in several placental mammals orders had led to propose that this structure appeared in an ancestor of placental mammals before the divergence of most groups and that was later lost in some species like mice (Dehay et al., 2015).

Regarding the genetic programs underlying cortical folding, several genes have been involved in different mechanisms and at different stages. Many of them were identified in people exhibiting cortical folding anomalies, such as polymicrogyria and lissencephaly. In fact, patients carrying mutations in genes such *LIS1, doublecortin* (*DCX*), and *cyclin-dependent kinase 5* (*CDK5*) show lissencephaly (Pilz et al., 1998; Kerjan and Gleeson, 2007; Magen et al., 2015). Genetic manipulations in animal models such as the ferret that displays a gyrencephalic brain, have allowed to show that in fact *CDK5* knockout in the ferret cerebral cortex *in vivo* impairs cortical folding (Shinmyo et al., 2017). Moreover, ferrets lacking *DCX* lack cortical folds (Kou et al., 2015). As mentioned before, genes affecting the generation and amplification of bRGCs are key factors in the formation of cortical folds. For instance, loss of function of the protein Trnp1 and activation of the SHH signaling pathways increased the number of bRGCs and led to the appearance of cortical folding in mice (Stahl et al., 2013; Wang et al., 2016). It has also been shown that extracellular matrix components such as HAPLN1, Lumican, and Collagen I induce folding of the cortical plate in human fetal neocortex explant systems suggesting that extracellular matrix components play a role in the folding of the human neocortex (Long et al., 2018).

On the other hand, it was early suggested that cortical folding is determined by hydraulic pressure from the cerebrospinal fluid and blood vessels acting on a limited cranial volume (Welker, 1990). Although these early theories were discarded due to the lack of experimental evidence, it has been suggested more recently that cortical folding results from internal or external biomechanical forces (Kroenke and Bayly, 2018). In fact, computational and mathematical models combined with experimental approaches have been developed in order to explain the biomechanical forces that govern folding. In order to simplify computational models the developing brain is represented before the emergence of sulci and gyri, as a structure consisting of two zones: the inner zone composed by the tissue between the cortical plate and the ventricle and the outer zone, conformed by the cortical plate (Kroenke and Bayly, 2018). Then, two main hypothesis have been proposed to establish if the mechanical forces inducing folding arise from the outer or the inner zone: (i) “buckling due to differential expansion” that proposes that the tangential expansion of the outer zone relative to the inner zone is the main force inducing folding (Xu G. et al., 2010; Bayly et al., 2014) and (ii) “axon tension” that suggests that such forces emerge from axons in the inner zone (Richman et al., 1975; Van Essen, 1997). Another theory has been recently developed to explain the expansion of supragranular layers in primates (Nowakowski et al., 2016). This theory, named “Supragranular Cortex Expansion Hypothesis,” proposes that primate cortical neurogenesis progresses in two stages. During early neurogenesis, basal fibers of ventricular radial glia contact the pial surface and newborn neurons migrate along ventricular as well as outer radial glia fibers. In late neurogenesis, newborn neurons reach the cortical plate only along outer radial glia fibers that do not contact the ventricular surface. In this second stage the scaffold formed by radial glia is broken and there is a discontinuous scaffold formed by two morphologically and molecularly distinct radial glia subtypes: ventral RG and outer RG. This model proposes that the tangential and radial expansion of the supragranular neuronal layers in primates is only dependent in neurogenic divisions of outer RG cells leading to a disproportionate expansion of supragranular cortex relative to infragranular cortex (Nowakowski et al., 2016).

Although these theories based on genetics or biomechanical forces into the determination of cortical folding appear to build upon contrasting ideas, a combination of early events determined by molecular genetic programs that set the cellular composition of the cortex and later events determined by the regional varying mechanical forces seem to better explain the appearance of gyri and sulci in the brain cortex of mammals.

Certainly, the impressive amount of knowledge that has accumulated in the last years related to mechanisms underlying cortical folding has shed light on the evolution of this salient characteristic unique to mammals. In fact, there is clear evidence that the most recent ancestor to all mammals already exhibited a gyrencephalic brain (O'Leary et al., 2013; Lewitus et al., 2014). Thus, it is possible to speculate that in the ancestor of all extant mammalian lineages there were already molecular mechanisms that make it possible to generate a gyrencephalic brain.

Definitely the availability of more comparative studies among vertebrates and new advances in technologies promise to render a better understanding of the evolution of this complex mammalian feature. Moreover, as it will be discussed below, several hominoid-specific genes have been recently linked to the regulation of cortical folding in humans.

### Interneurons Origin, Development, and Evolution

As mentioned before, during development the neocortex is populated by two main groups of neurons: excitatory projection neurons and inhibitory interneurons, that are mainly generated outside the cortex. In fact, inhibitory interneurons that mainly express GABA are originated in the medial and caudal ganglionic eminences and in the preoptic area and then migrate first tangentially in two streams over long distances into the cerebral cortex and then radially inside the cortex in order to become integrated into the various cortical layers (Buchsbaum and Cappello, 2019). The tangential migration of interneurons is regulated by multiple factors and although a deep review of them is not within the reach of this review, I will briefly mention some of the key factors involved in this important process of neocortical development. Excellent recent reviews on the matter are available (Faux et al., 2012; Hu et al., 2017; Lim et al., 2018). It has been shown that *connexin 43* and *Sox6* play important roles in the switch between tangential migration and radial migration (Azim et al., 2009; Batista-Brito et al., 2009; Elias et al., 2010). Another important factor controlling the correct path of migrating interneurons is the CXCL12/CXCR signaling pathway that seem to play a dual role, first attracting interneurons to the neocortex and then guiding their tangential migration until the correct radial signal is received (Faux et al., 2012). Once in the cortex, radial migration and lamination seem to be influenced by cues provided by pyramidal cells. Thus, *neuregulin 3* (*Nrg3*) expressed by pyramidal cells, facilitates the dispersion of cortical interneurons in the laminar dimension of the cortex (Bartolini et al., 2017). The correct lamination of interneurons in the CP is controlled by intrinsic and extrinsic factors. Among the extrinsic factors, reelin seems to also play a role in the layering of these neurons since abnormal lamination has been observed when reelin signaling is disrupted (Hevner et al., 2004; Hammond et al., 2006; Pla et al., 2006; Yabut et al., 2007). However, it is not clear if it is due to reelin signaling (Hammond et al., 2006) or to the location of pyramidal neurons (Pla et al., 2006). Among the intrinsic factors it has been suggested that the time of generation, the site of origin and also the cell-intrinsic genetic programs that they display influence not only on the final destination of interneurons in the cortex but also on the type of inhibitory cell that they become. Regarding the site of origin it has been suggested that interneurons arising from a common progenitor preferentially form clusters in the cortex (Brown et al., 2011; Ciceri et al., 2013) but this view has been recently challenged (Mayer et al., 2015). On the other hand, using single-cells transcriptome analyses, Mi et al. (2018) showed that shortly after the interneurons become postmitotic in their site of origin, their diversity is already evident due to the distinctive transcriptional programs that they display, and this transcriptional signature underlies their final differentiation in the developing cortex. Tangential migration by inhibitory interneurons from the subpallium to the pallium is a process highly conserved among vertebrates. There is evidence that suggests that the migratory pathways of neocortical GABAergic interneurons are mainly conserved among mammals (Tanaka and Nakajima, 2012). However, the site of origin may differ among species, because interneurons appear to be generated within the neocortex in addition to the ganglionic eminences in cynomolgus monkeys and humans (Letinic et al., 2002; Petanjek et al., 2009; Hansen et al., 2010; Jakovcevski et al., 2011; Yu and Zecevic, 2011). However, we are still far from understanding lineage-specific differences among mammals and vertebrates that can illuminate our knowledge about the complex mechanisms underlying interneurons development and evolution.

## Genetics Changes Underlying the Evolution of Mammals

### Birth of Mammals From a Genetics Perspective

I will review in the following sections the genetic changes that could have led to the appearance of the neocortex in mammals. However, beyond the comparative studies analyzing particular gene functions in mammals and other tetrapods it is important to note at this point that the study of genome-wide changes in the lineage leading to mammals that could underlie the emergence of mammals is still in its infancy.

In this regard, it has been found that in the lineage leading to Eutherian mammals 357 novel ancestral placental genes appeared *de novo* through different mechanisms including gene duplication and divergence (Dunwell et al., 2017). Of these, 41 novel genes are expressed in the brain suggesting that the emergence of new genes has contributed to the evolution of the mammalian brain. Focusing on particular groups of genes, Niimura and Nei (2005) found a striking expansion of a particular group of olfactory receptor genes in mammals suggesting that this type of genes contributed to particular characteristics of this group of vertebrates. Although duplication and divergence of existing genes are two widespread mechanisms for the appearance of new genes, the emergence of genes completely *de novo* has been shown to play an important role in the evolution of mammals. In fact, it has been found that several key mammalian genes have originated *de novo* from non-coding sequences (Luis Villanueva-Cañas et al., 2017).

Furthermore, another mechanism of *de novo* origin of functional sequences, involves transposable elements. In this regard, it has been demonstrated that some particular families of transposable elements have been the origin of gene regulatory sequences that control the expression of pre-existing genes in the mammalian lineage (Santangelo et al., 2007; Sasaki et al., 2008; Franchini et al., 2011). Alongside, comparative genomics analyses have allowed to detect not only coding but also non-coding regions that evolved a higher rate in the therian mammalian lineage (Holloway et al., 2016). Actually, 4,797 accelerated regions, principally non-coding have been identified and it has been proved that several of them behave as transcriptional enhancers that gained function in mammals compared to the orthologous region in non-mammalian vertebrates. Altogether, these data suggest that mammals underwent extensive remodeling of their genome that led to the acquisition of novel genes and novel expression patterns that probably underlie the evolution of morphological and functional novelties that characterize them. However, since no specific genes or regulatory regions have been identified so far related to the acquisition of the six-layered neocortex, more bioinformatics and functional studies will be necessary to identify which genes underlie the evolution of this mammalian novelty.

### Genetic Pathways Underlying Mammalian Brain Development and Evolution

To start unraveling the history of the genetic pathways that could underlie the evolution of the mammalian neocortex we need first to understand some of the genetic mechanisms that are in place during neocortex development. Thus, I will present in this section evidence from comparative studies that can help us to understand how changes in genetic mechanisms could have determined the evolution of the six-layered mammalian neocortex. There are several genetic pathways that are responsible for the development of the neocortex in mammals (Table 1). These pathways participate in the three different processes that are key during cortex development: neurogenesis, neural migration, and maturation.

**Table 1:** Signaling pathways involved in brain development and evolution.

**Pathway**	**Functions**	**Reported species**	**References**
Wnt/b-catenin	- Controls precursor population	Mouse	Chenn and Walsh, 2002; Logan and Nusse, 2004
Fibroblast growth factors	- Regulate neurogenesis in the developing cortex	Mouse, human, ferret	Raballo et al., 2000; Korada et al., 2002; Storm et al., 2006; Rash et al., 2013; Masuda et al., 2015; Heng et al., 2017; Matsumoto et al., 2017
Bone morphogenetic proteins	- Induce patterning of the telencephalon - Promotes RGCs differentiation	Mouse	Li et al., 1998; Bond et al., 2012
Sonic hedgehog	- Control the number of bRGCs and IPCs - Induce cortical folding	Mouse, human	Fuccillo et al., 2004; Dorus et al., 2006; Xu Q. et al., 2010; Baudoin et al., 2012; Wang et al., 2016; Yabut et al., 2020
Notch	- Represses proneural genes (Mash1, Ngn2, and Math1) - Maintains RGCs stemness	Human, mouse, chicken, gecko	Kageyama et al., 2008; Nomura et al., 2013a
Robo-Slit	- Generation and migration of cortical interneurons and pyramidal neurons	Mouse, chicken, snake	Andrews et al., 2006; Hernandez-Miranda et al., 2011; Zheng et al., 2012; Gonda et al., 2013; Yeh et al., 2014
Reelin	- Controls radial migration and laminar positioning of pyramidal neurons in the cortical plate	Human, mouse, turtles, crocodiles, lizards and birds	D'Arcangelo, 2005; Cabrera-Socorro et al., 2007; Nomura et al., 2008; Meyer, 2010
Transcription factors and transcriptional regulation	- Influence the differentiation of functional regions of the cortex - Control proliferation, differentiation and migration of cells in the cortex	Human, mouse	Nord et al., 2015; Ypsilanti and Rubenstein, 2016

#### Wnt/b-Catenin Signaling Pathway

The canonical Wnt signaling pathway plays a key role during brain development (Harrison-Uy and Pleasure, 2012). Wnt proteins act on target cells through the binding to a receptor complex [Frizzled (Fz)/low density lipoprotein (LDL) receptor-related protein (LRP)] that is located at the cell surface of apical progenitors in the developing cortex. Ligand binding induces stabilization of the cytoplasmic b-catenin, which levels are regularly kept low as a consequence of the degradation triggered by its phosphorylation mediated by GSK3b (Logan and Nusse, 2004). Thus, when a cell receives Wnt, this signals triggers the inhibition of the degradation pathway, and as a consequence β-catenin is stabilized and translocates into the nucleus to associate to TCF/LEF transcription factors, which trigger the transcription of downstream effectors (Logan and Nusse, 2004). It has been shown that transgenic mice expressing a stabilized form of beta-catenin in neural precursors develop enlarged brains and display an increase in cerebral cortical surface area and the appearance of folds mirroring sulci and gyri (Chenn and Walsh, 2002). However, it has been lately argued that the folding observed in this mouse model do not represent authentic gyrencephaly that normally affects only the pial surface but not the ventricular surface, whereas the folding observed by Chenn and Walsh affected both, the pial and the ventricular surface (Borrell, 2018).

#### Fibroblast Growth Factor Signaling

Fibroblast growth factor (FGF) ligands constitute a family of peptides that act both intracellularly and through secretion into the extracellular space. There have been described 22 FGFs so far and at least 13 have been shown to be expressed in the CNS throughout development (Fgf1,2, 3,7,8, 9,10,13,15,16,17,18,22) in particular areas of the neuroepithelium (Agirman et al., 2017). FGF ligands bind to their receptor FGFRs that are located in the cell membrane. So far four receptors have been described and three of them, FGFR1, FGFR2, and FGFR3 are expressed in the developing brain. It is now known that FGF signaling is critical for the regulation of neurogenesis in the developing cortex. In fact, it has been shown that the deletion of the *Fgf2* gene decreased the number of glutamatergic excitatory neurons resulting in a reduced anterior neocortex (Raballo et al., 2000; Korada et al., 2002). In addition, it has been shown that mice with impaired *Fgf8* gene expression display reduced proliferation and increased levels of apoptotic cells in the developing telencephalon (Fukuchi-Shimogori and Grove, 2001; Garel et al., 2003; Storm et al., 2006). It has been suggested that FGF signaling is key to the expansion of the SVZ. In fact, it has been reported that increased FGF signaling expands the generation of IPs without affecting bRGCs and leads to gyri formation in the rostrolateral developing forebrain (Rash et al., 2013). In addition, it has been shown that Erk-FGF signaling is more important in human RGCs compared to mouse RGCs since increasing Erk-FGF signaling in mice leads to the generation of bRGCs population without inducing folding in the neocortex (Heng et al., 2017). On the other hand, it has been revealed that increasing FGFs signaling into the ferret cerebral cortex through *in utero* electroporation, leds to an increase in the number of undulating folds, suggesting that an excess of FGF signaling is sufficient to induce the appearance of additional cortical folds (Masuda et al., 2015). Moreover, suppression of FGF signaling completely through the use of a dominant negative form of one of the FGF receptors, impairs cortical folding in the ferret showing that FGF signaling is required for cortical folding (Matsumoto et al., 2017). In addition, blocking FGF signaling reduces the proliferation of oSVZ progenitors. This evidence indicates that FGF signaling is critical for cortical folding in gyrencephalic mammals and is a key upstream regulator of the production of oSVZ progenitors (Matsumoto et al., 2017).

#### Bone Morphogenetic Proteins

Bone morphogenetic proteins (BMPs) are constituents of the transforming growth factor β (TGF-β) superfamily (Derynck and Zhang, 2003; Shi and Massagué, 2003; Miyazono et al., 2010). BMPs bind to heterotetrameric complexes that consist of pair type I/II receptors and co-receptors and activation of these complexes results in the phosphorylation of particular cytoplasmic SMAD proteins that translocate to the nucleus to initiate transcriptional activity (Bond et al., 2012). BMP2, 4, 5, 6, and 7 are secreted by the cortical hem and interact with Wnts to induce the dorsomedial patterning of the telencephalon (Bond et al., 2012). BMP2 and BMP4 are the main participants of the BMP signaling in the developing cortex (Shakèd et al., 2008). Previous studies reported that BMP signaling promotes the neuronal differentiation of RGCs (Li et al., 1998). In addition, more recently it has been shown that the null mutation of the *Foxg1* gene generates hypoplasia of the mouse telencephalon and loss of ventral telencephalic structures (Martynoga et al., 2005). In these mice it is observed that excess neurons are produced leading to the depletion of the progenitor pool and constraining the growth of the telencephalon. These effects are mediated by the regulation of FGF and BMP signaling pathways (Martynoga et al., 2005). Although the key role of this signaling pathway is noticeable, a lack of comparative studies among mammals and other non-mammalian vertebrates prevent us from driving conclusions about the importance of this pathway in the evolution of the mammalian neocortex.

#### Sonic Hedgehog

Sonic hedgehog (Shh) is a diffusible secreted protein that belongs to the hedgehog family composed by two other members: Indian hedgehog (Ihh), and Desert hedgehog (Dhh) (Echelard et al., 1993; Roelink et al., 1994). In the developing forebrain, Shh is mostly secreted from the ventral telencephalon into the cerebro-spinal fluid (Ericson et al., 1995). In addition, it is also produced by Cajal-Retzius cells in the marginal zone (MZ) of the cerebral cortex, by the choroid plexus and by the interneurons that migrated to the cortical plate (Komada et al., 2008; Huang et al., 2010). Shh mediates its action via a receptor complex composed of two transmembrane proteins: Patched (Ptch1) and Smoothened (Smo) (Corbit et al., 2005; Rohatgi et al., 2007). Smo is a G-coupled protein that activates a complex signaling pathways that includes the activation of the Gli family (Gli1, Gli2, and Gli3) of transcription factors (Sasaki et al., 1999) that in turn activated among others the transcription factor Nkx2.1 that is required for the proper specification of specific interneuron subtypes (Butt et al., 2008). Besides, ectopic activation of Shh signaling in neocortical progenitors increase expression of FGF15, leading to the activation of FGF and MAPK signaling pathways and triggers the expression of ventral forebrain progenitors typical genes (Yabut et al., 2020). In the ventral telencephalon, Shh signaling plays a key function in the production of GABAergic interneurons, which later colonize the cortical plate by tangential migration (Fuccillo et al., 2004; Xu Q. et al., 2010; Baudoin et al., 2012). In contrast, a more limited Shh signaling has been described in the developing cortex where its function is still poorly understood. However, it has been recently shown that the constitutively activation of Shh signaling in mice increased the number of bRGCs and IPCs and induced folding in the lissencephalic mouse neocortex, whereas the loss of Shh signaling reduced the number of bRGs and IPCs and neocortical size (Wang et al., 2016). In addition, it has been found that SHH signaling was greatly active in the human fetal neocortex whereas in the mouse embryonic neocortex Shh signaling displayed a reduced activity. Moreover, blocking SHH signaling in human cerebral organoids decreased the number of bRGs. These findings led the authors to propose that the strong SHH signaling observed in the human fetal neocortex may have contributed to bRGC and IPs expansion leading to neocortical growth and folding (Wang et al., 2016).

It has been reported that the molecular evolution of the gene SHH is dramatically accelerated in primates relative to other mammals. Within primates, the acceleration is most noticeable in the lineage leading to humans (Dorus et al., 2006). These results suggest that SHH underwent molecular changes under positive selection in the lineage leading to humans and this is interesting considering that the loss of one functional copy of SHH in humans leads to serious neurological and craniofacial developmental problems (Nanni et al., 1999) whereas the loss of one copy of SHH in mice does not induce appreciable developmental abnormalities (Chiang et al., 1999).

#### Notch Signaling

Notch receptors are transmembrane proteins composed of an extracellular EGF-like domain that bind ligands and an intracellular domain that after a series of modifications translocates into the nucleus. In fact, ligand binding triggers enzymatic events that result in cleavage of the intracellular domain that carries nuclear localization signals that guide it into the nucleus (Stifani et al., 1992; Schroeter et al., 1998; Struhl and Adachi, 1998). There are five Notch receptors and five canonical ligands belonging to the Jagged (Jag1 and Jag2) or Delta-like (Dll1, Dll2, Dll4) families (Zhang et al., 2018). In the developing cortex, the Notch signaling pathway is critical in regulating cortical neurogenesis. RGCs express Notch1 and Notch3 receptors and the ligands are expressed by neighboring neurons or IPs. After the ligand binds the Notch receptor, it experiences two successive cleavages, the first one is driven by the disintegrin/metalloprotease ADAM10 and the second one is performed by the γ-secretase and results in the release of the extracellular domain and the Notch intracellular domain (NICD). Then, NICD translocates to the nucleus and binds to CBF1 or Rbpj co-factor to trigger the transcription of many genes, including the Hairy enhancer of split (Hes) genes. Hes are transcription factors of the basic-helix-loop-helix (bHLH) family that repress the expression of proneural genes such as such as Mash1, Ngn2, and Math1, and ensure that RGCs preserve stemness (long-lasting progenitor potential) (Kageyama et al., 2008).

Comparative studies using a reptile model species (gecko), chicken and mouse have shown that Notch signaling is activated at different stages and in a species-specific manner in the developing cortex (Nomura et al., 2013a). In fact, using a Notch responsive reporter vector the authors show that geckos exhibit higher Notch activity particularly at later embryonic stages compared to mouse and chicken (Nomura et al., 2013a). These results suggest that the spatio-temporal regulation of Notch signaling in neural stem/progenitor cells could constitute the molecular mechanism underlying the inter-species differences observed in pallial neurogenic rates. These findings led the authors to hypothesize that changes in the regulation of neural stem/progenitor cells, including Notch signaling activation mechanisms, arose independently in the ancestors of mammals and archosaurs (Nomura et al., 2013a). Then, additional changes in the proliferation of apical progenitors and the emergence of basal progenitors might have contributed to the expansion of neurogenesis that characterizes the cerebrum of birds and mammals (Nomura et al., 2013a). Of note, it is important to mention that the Notch pathway underwent also species-specific changes in the human lineage (see below) supporting this hypothesis that pinpoint to the Notch pathway as a key player in the evolution of the neocortex in different non-mammalian and mammalian lineages.

#### Robo-Slit Signaling

The Roundabout (Robo) family of receptors together with their ligands, the Slit proteins, are abundantly expressed in the developing forebrain and play critical roles in the generation and migration of cortical interneurons (Andrews et al., 2006; Hernandez-Miranda et al., 2011) and also pyramidal neurons (Yeh et al., 2014). It has also been shown that Robo1 and Robo 4 play a role in radial migration of pyramidal neurons (Zheng et al., 2012; Gonda et al., 2013).

It has been recently shown that Robo1/Robo2 signaling plays a differential role between direct and indirect neurogenesis in the olfactory bulb (OB) vs. neocortical areas in mice (Cárdenas et al., 2018). Whereas, grows at a faster rate than the neocortex and this fast neurogenesis is due to higher direct neurogenesis in the OB. Double mutants for Robo1/Robo2 displayed impaired grow and development in the OB as a consequence of deficit in neurogenesis. Moreover, Slit1/2 double mutants showed the same defects observed in Robo1/2 mutants indicating that these are the receptors involved in neurogenesis in the OB. In order to understand which other pathways could be interacting with Robo-Slit signaling to control direct and indirect neurogenesis balance authors tested the Notch ligand Dll1 because it is expressed in a differential manner in the OB and the neocortex, showing lower levels in OB and higher in the neocortex. The authors found that *Dll1* levels in the OB are increased in Robo1/2 mutants suggesting that *Dll1* expression in the OB is downstream of Robo-Slit signaling. However, CRISPR/Cas9-mediated impairment of *Dll1* expression did not affect direct neurogenesis. Only the combination of overexpression of active forms of Robo1/Robo2 and reduction of *Dll1* expression led to increased direct neurogenesis in the neocortex. These authors show that in the chicken dorsal cortex a Robo1/2 signaling also plays a role in maintaining the balance between direct and indirect neurogenesis. In the African house snake they found that the only mode of division in the dorsal cortex is direct neurogenesis and that manipulation of Robo signaling and Dll1 levels led to reduced direct neurogenesis. These results led the authors to propose that an attenuation of Robo signaling in the neocortex during mammalian evolution led to the emergence of cortical basal progenitors and the SVZ and the blockade of direct neurogenesis. The authors also hypothesize that these changes combined allowed the expansion and complexification of the mammalian cerebral cortex (Cárdenas et al., 2018). Although the hypothesis is tempting the genetic mechanisms that led to a decrease in Robo1/2 expression in the mammalian neocortex need to be uncovered.

#### Reelin-Mediated Signaling Pathways

A striking difference between mammalian and sauropsids is the development of Cajal-Retzius (CR) cells (Figure 2). These cells are a special kind of neuron that is generated in the VZ located in the limit between dorsal and ventral telencephalon and also in the cortical hem. CR cells are the most significant source of reelin, an extracellular matrix glycoprotein essential for cortical development. CR cells migrate from their places of origin to the Marginal Zone (MZ) and through the secretion of Reelin they control radial migration and laminar positioning of pyramidal neurons of the cortical plate (Meyer, 2010). It has been shown that a mice mutant for the expression of reelin (reeler mouse) (for a review on this mutant see D'Arcangelo, 2005) displays a disorganized pattern of migration of neurons that result in an inverse layering of the cortex (reviewed by Aboitiz et al., 2002). Sauropsids like turtles, crocodiles, lizards and birds display scarce Reelin expressing cells in the telencephalic marginal zone during cortex development (Bernier et al., 1999, 2000; Goffinet et al., 1999; Bar et al., 2000; Tissir et al., 2003). This reduced Reelin expression apparently results from the lack of CR cells originated from the cortical hem or ventral pallium (Bielle et al., 2005; Cabrera-Socorro et al., 2007). It has been shown that the increase of Reelin expressing cells in the avian dorsal cortex through experimental manipulation modifies the RGC fibers organization and the patterns of neuronal migration, suggesting that the increase of Reelin signaling was a key step in the evolution of the mammalian neocortex (Nomura et al., 2008, 2009).

#### Transcription Factors and Transcriptional Regulation

In addition to the signaling pathways mentioned above, it has been shown that a plethora of transcription factors play key roles into the regionalization of the cortex and then in the proliferation, differentiation and migration of cells. In fact, several transcription factors that are expressed in graded antero-posterior and ventral-dorsal patterns influence the differentiation of functional regions of the cortex. For instance loss of function studies have shown that *CoupTF1, Emx2, Lef1, Lhx2, Pax6*, and *Sp8* control the correct patterning of the cortex (Ypsilanti and Rubenstein, 2016). In addition, several transcription factors such as *Tbr1, Tbr2, Pax6, Emx1, Emx2, Fezf2, Ngn1, Ngn2, and Satb2*, that control the differentiation of glutamatergic neurons have been described (Lai et al., 2013; Ypsilanti and Rubenstein, 2016). Several recent reviews have analyzed in-depth the role of transcription factors in the development of the mammalian cortex, thus here I will only mention some salient examples of key TF controlling cortical development. For instance, Tbr1 and Tbr2 are transcription factors of the T-box family that play a key role in the proliferation and differentiation of glutamatergic neurons. For instance, Tbr2 controls the expression of hundreds of direct target genes and in this way influences the proliferation and differentiation of IPs in the developing cortex (Hevner, 2019). Another key transcription factor is Pax6 that controls patterning, migration, differentiation and neurogenesis in the cortex. The role of this TF in the development of the neocortex has been extensively reviewed elsewhere (Ypsilanti and Rubenstein, 2016). Regarding the development of GABAergic interneurons, several key transcription factors such as *Dlx2, Dlx2*, and *Nkx2.1* have been reported. These TFs regulate the expression of many important genes and are master controllers of subpallial generation of interneurons (Nord et al., 2015). Regarding the role of TFs in the evolution of the neocortex, a few studies have explored this matter. A study analyzing comparatively TF networks in primates concluded that these pathways have been modified in a lineage-specific manner in the prefrontal cortex, suggesting that this could be a more widespread mechanism of brain evolution (Berto and Nowick, 2018). Although our understanding of the role of TFs in cortical development and evolution is still incomplete, the emergence of RNA-seq and epigenetic analysis techniques combined with the use of mutant mouse pedigrees is allowing us to understand better the gene regulatory pathways that are altered when a particular TF is absent. These techniques are also being used in non-mammalian vertebrates to analyze cortical development. In this way, we will soon have a better picture of the gene regulatory networks controlling cortex development in mammals and how these networks evolved in vertebrates to render the evolution of the six-layered neocortex.

## Mammals, Brains Diversity and the Explosion of Behavioral Complexity

### The Diversity of Mammalian Brains

Mammals display a high diversity of brains and in the same mammalian order is frequent to find lissencephalic and gyrencephalic species (Figure 1). One interesting question is: which differences in developmental mechanisms in the neocortex underlie the cortical expansion observed in some mammals? As mentioned before, comparative studies among some model mammalian species are helping us to understand which cellular and molecular changes observed in the SVZ are correlated with changes in neural number and neural complexity. In the section below, I analyze current knowledge about the brains of different mammalian lineages that display the largest expansion of the neocortex.

### Big Brained Mammals: Elephants, Cetaceans, and Primates

**Figure 3:**
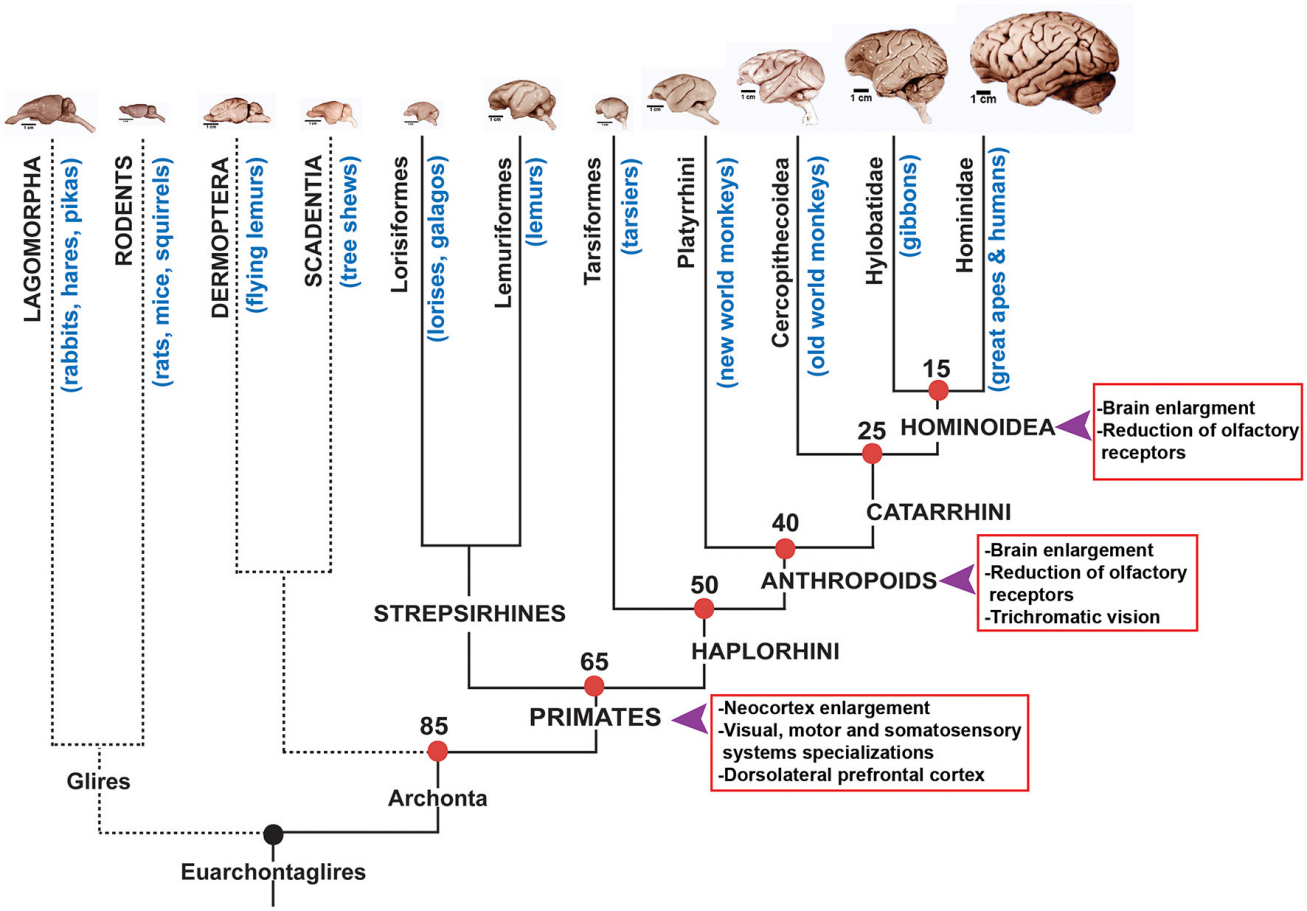
Phylogenetic tree of primates and related mammalian orders. On the top row representative brains of the different groups that composed the Euarchontoglires clade are shown. Primate groups and approximate times of divergence are indicated on the tree. The arrows indicated moments in history where brain volume has increased in the Anthropoid lineage according to Goodman (1999). Brain pictures are approximately at scale and are from the Comparative Mammalian Brain Collection (http://neurosciencelibrary.org) from the University of Wisconsin and Michigan State Comparative Mammalian Brain Collections, as well as from those at the National Museum of Health and Medicine funded by the National Science Foundation, as well as by the National Institutes of Health.

There are three lineages among placental mammals that display greatly enlarged brains: proboscidea that group elephants, cetaceans that assemble dolphins and whales and primates that include prosimians, monkeys, great apes and humans (Figures 1, 3).

#### Elephants

Elephants carry the largest brains of all terrestrial animals, and display the greatest cerebral cortex (Hart and Hart, 2007). Although elephants are capable of high order brain functions such as long-term memory, they are less able than Hominids like the chimpanzee in mirror self recognition or tool use. It has been recently found that the African elephant (*Loxodonta africana*) brain, which is about three times larger than the human brain contains 257 billion neurons, three times more neurons than the human brain but, the majority of these neurons (97.5%) are located in the cerebellum. On the other hand, the cerebral cortex which has twice the volume of the human cortex carry 5.6 billion neurons which represents one third of the neurons found in the human cerebral cortex (Herculano-Houzel et al., 2014).

#### Cetaceans

Cetaceans are a group of mammals that share a common ancestor with Artiodactyla and that conquered aquatic environments ~60 mya (Thewissen et al., 2001). Today members of this order inhabit oceans and rivers, they are mainly predators and are characterized by long living periods, a dedicated offspring care system and a complex social organization (Marino, 2007, book). In addition, this group is distinguished by big brains, behavioral complexity and salient cognitive capacities (Marino, 2007; Marino et al., 2007). The brains of cetaceans are very large in both absolute and relative size and possess encephalization quotients (EQ) that are second only to humans (Marino, 1998). Actually, the largest brain on earth belongs to the sperm whale which can reach up to 8,000 cubic centimeters. Some cetaceans evidence some of the most sophisticated cognitive abilities among all mammals and show impressive convergence in terms of cognition with primates, including humans. In fact, cetaceans display complex social behavior such as alliances (Connor, 2007) and cultural transmission of information including hunting techniques (Allen et al., 2013). In addition, they show elaborated communication systems that include complex vocalizations and mimicry (Ridgway et al., 2012; Sayigh, 2014). It has been suggested that in cetaceans, brain size predicts the magnitude of social and cultural behaviors observed in this group of aquatic mammals (Fox et al., 2017). The brains of modern cetaceans are different in several aspects to other mammalian brains and also to their mammalian ancestors. Their brains are characterized by a great expansion of the cerebral hemispheres and auditory structures, and reduction of olfactory areas (Marino et al., 2007). The neocortex of cetaceans is characterized by lacking layer IV, so in contrast to other mammals instead of having six well-defined layers, cetaceans possess five layers. This change has important implications for the distribution of afferent connections to the neocortex (Marino et al., 2007). In addition, it has been shown that the frontal lobe is reduced in cetacean brains in clear contrast to the enlargement of this region in primates (Morgane et al., 1980). It has been recently shown that cetaceans display in their cortices Von Economo neurons (Hof and Van Der Gucht, 2007; Butti et al., 2009). This type of neurons have been also described in humans, great apes (Allman et al., 2005, 2010) and elephants (Hakeem et al., 2009) and have been associated with certain aspects of higher cognitive abilities in humans such as social and emotional cognition, awareness, and intuition (Allman et al., 2005). It has been suggested that Von Economo have appeared convergently in phylogenetically unrelated groups of mammals like cetacean, hominids and elephants possibly under similar selective pressures that targeted specifically the evolution of cortical regions involved in complex cognitive and social-emotional capacities (Butti et al., 2009).

However, the lack of comparative gene expression studies in cetaceans and elephants prevents us from making any analyses about the gene and genetic pathways that could be involved in the evolution of the complex and marvelous elephant and cetaceans brains.

#### Primates

Primates emerged around 80–60 mya and then diversified in several groups that today are represented by more than 300 species (Figure 3). Primates have adapted to varied environments and ways of living and their brains show not only differences in size but also adaptations to different survival strategies. Primates display unique anatomical aspects compared to other mammals (Preuss, 2007; Kaas, 2013) and they also show differences in the way neurons and non-neuronal cells are packed in their brains (Herculano-Houzel et al., 2007). In addition, the neocortex in primates display much more functional areas subdivisions than non-primates. Thanks to the detailed analyses of prosimians (Strepsirrhine) it has been found that primates possess several cortical areas that are different compared to non-primates. One of the most distinctive characteristics of primates is their visual system, beyond the evolution of trichromatic vision that probably occurred in the Anthropoid lineage, it is also noticeable the frontal location of eyes which modified how information travels to the brain (Striedter, 2005). In the cortex, the primary visual area V1 is shared with all mammals but in primates it has specializations regarding connections and layering compared to non-primate mammals (Preuss, 2007). In addition, this primary visual cortex has two different processing modules and are dedicated to processing color information and orientation of the stimulus (Preuss et al., 1999; Kaas, 2012a). Besides, two other visual areas in the cortex (V2 and V3) also process visual information and show specializations in primates (Kaas, 2012b, 2013). Particularly it has been postulated that V3 is unique to primates and that a similar area that has been found in carnivores evolved independently (Kaas, 2012b). Comparisons between primate and non-primate brains indicate that the motor system is more complex and displays a higher number of premotor areas (9 or more) than non-primates that only have two to four (Wu et al., 2000). It has been shown that primates have a ventral premotor area that is involved in arm and mouth movements and that could be related to increased dexterity in primates (revised in Striedter, 2005). In addition, in primates it has been observed an increase in the number of somatosensory areas of the cortex that seem to be involved in touch sensitive fingertips and movement control (revised in Preuss, 2007).

**Figure 4:**
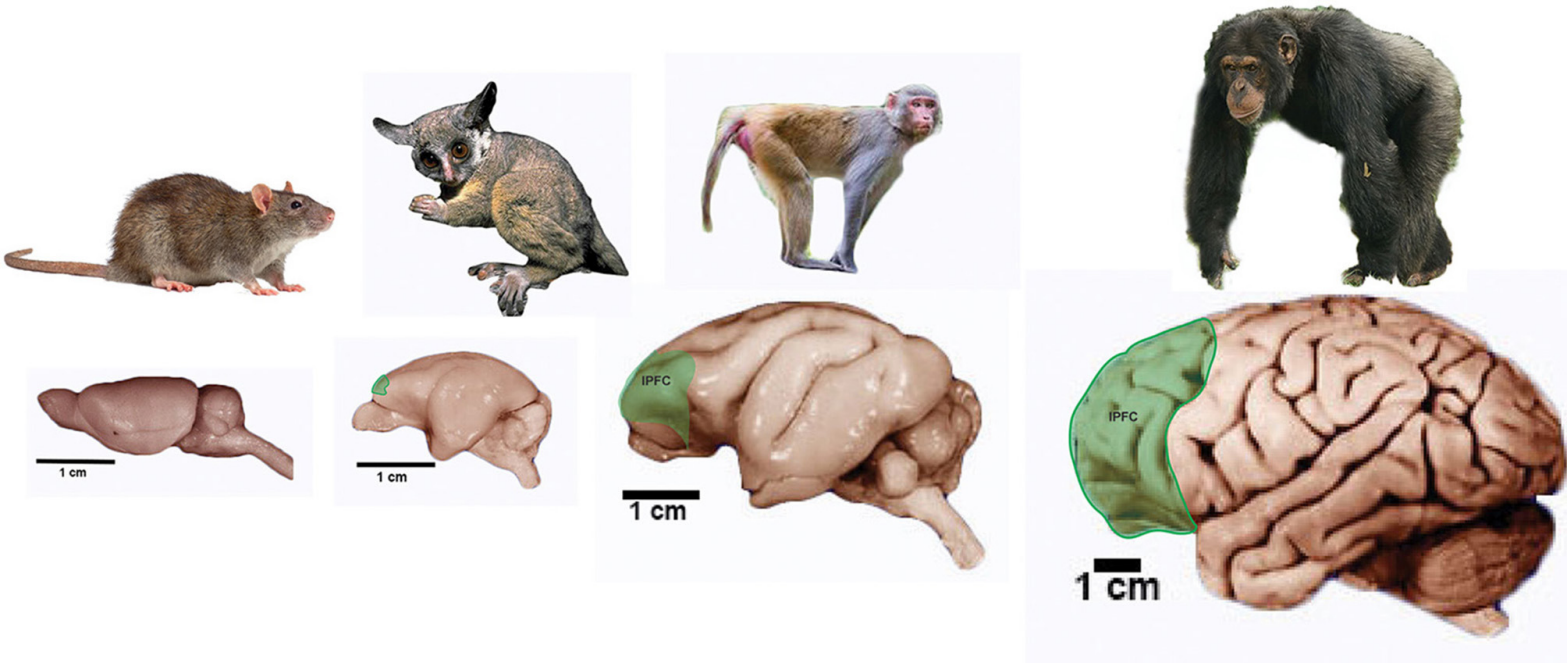
Prefrontal cortex in primates. Pictures of representative primate groups and the rat show the approximate location of the lateral Prefrontal Cortex (lPFC). Brain pictures are approximately at scale and are from the Comparative Mammalian Brain Collection (http://neurosciencelibrary.org) from the from the University of Wisconsin and Michigan State Comparative Mammalian Brain Collections, as well as from those at the National Museum of Health and Medicine funded by the National Science Foundation, as well as by the National Institutes of Health.

As well, primates show a great development of an area located in the frontal lobe that has been related to higher order cognitive abilities such as decision making: the prefrontal cortex (Figure 4). Even though non-primate mammals do have a prefrontal cortex it seems to be composed of only two regions whereas primates display three regions: the orbital prefrontal region, anterior cingulate or medial region (these two are present in non-primate mammals) and the dorsolateral or granular prefrontal cortex which seems to be unique to primates (Preuss, 1995, 2007; Striedter, 2005). Although there is some controversy about the dorsolateral prefrontal cortex being a primate innovation (Preuss, 2007) it is clear that this area is related to complex and flexible behaviors that are impaired when this area is damaged (Striedter, 2005).

Among primates, Apes displays a great enlargement of brain size and also a complex behavioral repertoire. Apes include the lesser apes with gibbons and siamangs and the great apes that include us, gorillas, chimpanzees, bonobos and orangutans. Compared to other primates, apes and humans (Hominoids) display larger brains, longer developmental periods, high energy requirements, lower reproductive rates and longer periods of parental care (Kaas, 2007, 2008, 2013).

Besides, it has been shown that the prefrontal cortex areas enlarged and became specialized during hominid evolution (Semendeferi et al., 2001). More recently it has been reported that human and great ape brain evolution is defined by the non-allometrically derived changes in cortical organization that include the extraordinary expansion of the prefrontal cortex (Smaers et al., 2017). It has been postulated that these changes in the prefrontal cortex underlies the increase in executive functions that characterize great apes and particularly humans and that are operated through this cortical region (Smaers et al., 2017).

## The Human Brain

### Genetic Basis Underlying the Evolution of the Human Brain

The human brain is a typical mammalian brain since it displays the six-layered neocortex with a well-developed SVZ. It has also the typical features of a primate brain such as a remarkably large neocortex including a large visual cortex and a lateral prefrontal cortex (Striedter, 2005; Preuss, 2007; Kaas, 2013). In spite of these overall similarities, our brain has a number of features that make it unique. In fact, the development and anatomy of our brain differentiate in various critical aspects from those of other primates. For instance, the human brain has the largest number of neurons of any primate since it carries ~86 billion (Azevedo and Carvalho, 2009) compared with an estimated number of neurons in chimpanzee and gorilla brains of 28 and 33 billion neurons, respectively (Herculano-Houzel and Kaas, 2011). However, as described above, the human brain is not the largest on Earth, being eclipsed by the giant brains of elephants and cetaceans (Roth and Dicke, 2005; Hart and Hart, 2007; Marino, 2007). It has been calculated that 20.9% of all neurons in the human brain are located in the cortex, which is more than 10% greater than the proportion of cortical neurons in any other mammal (Herculano-Houzel, 2012). Although it is hotly debated whether our neocortex is particularly unique compared to chimpanzee (Barton and Venditti, 2013a,b; Smaers, 2013; Smaers et al., 2017), it is clear that the human cortex contains the most neurons (16/18 billion) and is proportionally the largest (84% of the entire brain mass) of any mammal (Herculano-Houzel, 2009, 2012; Herculano-Houzel et al., 2014).

Besides displaying the largest numbers of neurons the human brain is unique in several other aspects. In fact, post-mortem studies showed that our brain displays distinctive features in terms of cellular and histological organization of the cerebral cortex (Sherwood et al., 2008; Preuss, 2010; Miller et al., 2012). In addition, the use of diffusion-tensor imaging, a non-invasive brain imaging technique, allowed to study comparatively long-range interactions in the cortices of human, macaque and chimpanzee brains and revealed outstanding differences in cortical connections (Rilling et al., 2008).

**Figure 5:**
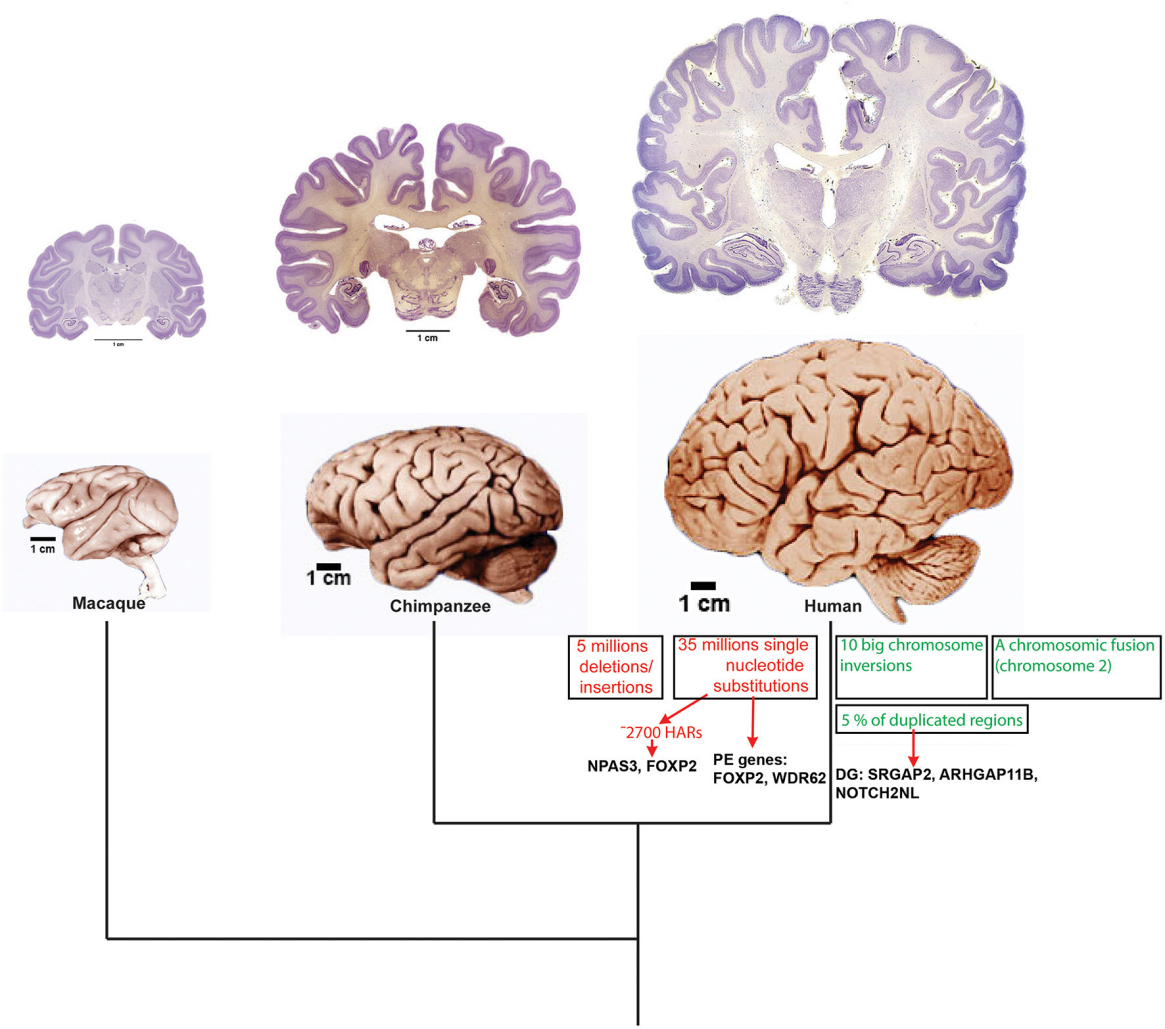
Genetic changes underlying human nervous system evolution. A schematic phylogenetic tree shows the relationships among macaque, chimpanzee and human. Above that brain pictures show a detail of the size differences among these three primate species. Brains are shown at scale. On top of that, brain coronal sections at the forebrain level show anatomic differences among the species. It is appreciated the great development of the gyrification in the three species. Brain pictures are approximately at scale and are from the Comparative Mammalian Brain Collection (http://neurosciencelibrary.org) from the from the University of Wisconsin and Michigan State Comparative Mammalian Brain Collections, as well as from those at the National Museum of Health and Medicine funded by the National Science Foundation, as well as by the National Institutes of Health. On the lineage leading to humans some salient genetic changes that have been uncovered in the last years are indicated. PE, positively selected genes; DG, duplicated genes.

However, in order to disentangle the evolution of humans' higher order cognitive abilities, such as abstract thinking, long term planning and an exceptional capacity to generate a complex language, we need first to address two challenging questions. The first is how to associate human cognition to particular neuroanatomical differences including brain size, number of neurons and a highly developed cortex. For instance, the neurobiological bases underlying our capacity to produce and elaborate language are not comprehensively understood, because surprisingly the essential areas controlling language in our brain are also present in chimpanzees (Cantalupo and Hopkins, 2001; Taglialatela et al., 2008). The second question is: how to link DNA changes to uniquely human neurobiology? (Figure 5). However, in the last two decades some progress has been made toward understanding the genetics underlying one of the most distinctive human cognitive traits: our spoken language (Vallender et al., 2008; Scharff and Petri, 2011; Preuss, 2012; Fisher, 2019). Nevertheless, we still know very little about how these genetic differences impact into molecular, cellular and anatomical mechanisms to shape the distinctive features of the human brain. Several attempts have been carried out to identify the genetic differences that could underlie the evolution of the human brain and many human-specific DNA sequences have been identified (Figure 5). After the sequencing of the human genome (Lander et al., 2001; International Human Genome Sequencing Consortium, 2004) as well as countless other mammalian genomes, including those of the macaque and the chimpanzee (Chimpanzee Sequencing Analysis Consortium, 2005; Rhesus Macaque Genome Sequencing Analysis Consortium et al., 2007), we have the availability of numerous genome-wide catalogs of human-specific genome changes that include genes that underwent positive selection in humans, genes displaying human-specific differences in splicing, chromosome segmental duplications that resulted in the appearance of new human genes and evolutionarily conserved non-coding sequences carrying human-specific mutations (reviewed in Sikela, 2006; Vallender et al., 2008; O'Bleness et al., 2012; Hubisz and Pollard, 2014; Bae et al., 2015; Silver, 2016; Franchini and Pollard, 2017; Sousa et al., 2017). The challenge that scientists of this century face is to connect human-specific genetic differences to unique human traits.

#### Gene Duplication and Gene Loss

The discovery of human-specific genomic variants began prior to genome sequencing. In fact, the use of chromatin-stained banding techniques allowed identification of the fusion of two ancestral hominid chromosomes that gave rise to human chromosome 2 and pericentric inversions on chromosomes 1 and 18. In addition, this technique uncovered the existence of human-specific constitutive heterochromatin C bands on chromosomes 1, 9, 16, and Y (Yunis and Prakash, 1982). Large genomic deletions, duplications, and rearrangements are relatively rare, but due to their size, that could usually be thousands of base pairs, they frequently encompass many developmental loci and have a large impact on gene and phenotype evolution (Girirajan et al., 2011, 2013; Coe et al., 2014). Thanks to the use of techniques such as fluorescent *in situ* hybridization (FISH) and comparative genomic hybridization (CGH) arrays it has been possible to identify more than 60 human-specific segmental duplications (Jauch et al., 1992; Goidts et al., 2006) and 152 genes displaying copy number variation (Fortna et al., 2004; Armengol et al., 2010). A significant amount of these structural variants have altered gene expression inducing phenotypical changes in humans. For instance, the pericentric inversion of chromosome 1, has been linked to neurogenetic diseases in humans and contains copy number variations of several developmental genes including HYDIN (Doggett et al., 2006), SLIT-ROBO Rho GTPase-activation protein (SRGAP2) (Dennis et al., 2012), and genes containing DUF1220 domain protein such as the neuroblastoma breakpoint family (NBPF) (Fortna et al., 2004; Dumas and Sikela, 2009). Thus, two rounds of human-specific duplication of the locus created four copies of the gene *SRGAP2*: *SRGAP2A, SRGAP2B, SRGAP2C*, and *SRGAP2D* (Dennis et al., 2012). In addition, it has been shown that SRGAP2C is expressed throughout and development and in the adult human brain (Charrier et al., 2012). It was also found that SRGAP2C dimerizes with the ancestral SARGAP2A and inhibits its function. It had been previously shown that the ancestral copy of *SRGAP2* reduces the rate of neuronal migration and leads to a lesser amount of cells in the cortical plate (Guerrier et al., 2009). On the other hand the action of SRGAP2C inhibits this process and leads to an increased rate of migration (Charrier et al., 2012). In addition, SRGAP2C retards dendritic spines maturation in neurons. These results prompted the authors to suggest that the appearance of human-specific paralos of SRGAP2 contributed to the evolution of some features of the human brain (Charrier et al., 2012).

A distinct human-specific structural variant occurred at chromosome 15q13-q14 and resulted in the duplication of several genes, including *ARHGAP11B*, which is a partial copy of the gene *ARHGAP11A* (Antonacci et al., 2014). *ARHGAP11B* appeared on the human evolutionary lineage after the divergence from the chimpanzee. In addition, the duplication of *ARHGAP11B* predates the split of our lineage with those of archaic humans since this gene is also found in Neanderthals and Denisovans. *ARHGAP11B* was identified as one of the exclusively expressed genes in human basal and apical radial glia compared to neurons in a transcriptomic analysis of the fetal human neocortex (Florio et al., 2015). To explore the function of *ARHGAP11B* in corticogenesis, Florio et al. expressed *ARHGAP11B* in mouse neocortex by in utero electroporation on embryonic day 13.5 (E13.5) (Florio et al., 2015). This led to an increase in basal but not apical mitoses and the expansion of Tbr2-expressing basal progenitors at E14.5. In turn, this increase in basal progenitors led to thickening of the SVZ. On the other hand, overexpression of *ARHGAP11A* did not increase basal progenitors. Furthermore, in half of the cases analyzed, *ARHGAP11B* expression induced at E13.5 resulted in neocortex folding at E18.5, in the otherwise smooth mouse neocortex. These mice showed an increase of cortical plate area in the gyrus-like structures compared with the contralateral smooth neocortex that displayed normal cortical lamination (Florio et al., 2015). In addition, it has been shown that ARHGAP11B displays a truncated GAP domain which is responsible for RhoGAP activity in ARHGAP11A (Florio et al., 2015). In fact, a single C→ G base change in exon 5 in the *ARHGAP11B* gene generated a novel GU-purine splice donor site that induces the deletion of 55 nucleotides through mRNA splicing leading to the GAP domain truncation and addition of a human-specific carboxy-terminal amino acid sequence (Florio et al., 2016) which is unique to ARHGAP11B since it has not been yet found in any other protein described in the animal kingdom (Florio et al., 2015). It has been hypothesized that this novel domain has a key role in basal progenitors amplification induced by ARHGAP11B (Florio et al., 2016). Regarding the function of ARHGAP11B, it has been recently shown that this protein is localized in the mitochondria in contrast to ARHGAP11A which is found in the nucleus (Namba et al., 2020). In the mitochondria, this protein interacts with the adenine nucleotide translocase (ANT) and inhibits the mitochondrial permeability transition pore (mPTP), apparently leading to an increase in calcium concentration as well as an increase in glutamine-dependent mitochondrial respiration (Namba et al., 2020). This mitochondrial metabolic pathway is key for the increase in basal progenitors mitotic levels mediated by ARHGAP11B (Namba et al., 2020).

In order to study ARHGAP11B in gyrencephalic mammals, this gene was also introduced into ferret embryos, ferrets are gyrencephalic mammals that display an expanded neocortex and constitute more suitable models to study brain evolution and development in gyrencephalic brains (Kalebic et al., 2018). This manipulation increased proliferative basal radial glia number and resulted in extension of the neurogenic period leading to increased neuron density in the upper cortical layers (Kalebic et al., 2018). More recently, the generation of genetically modified common marmosets carrying *ARHGAP11B* under control of the human promoter of this gene that directs its expression to the developing neocortex increased the number of basal RGCs in the oSVZ of this lissencephalic primate leading to increased numbers of upper-layer neurons and induced an enlargement of the neocortex that resulted in cortical folding (Heide et al., 2020).

In addition, the recent description of NOTCH2 human-specific paralogs suggest that progenitor proliferation and neuronal differentiation pathways have been modified in the human lineage. Two recent works found independently that the gene chromosome region where the gene NOTCH2 is located in the human genome (1q21.1) underwent a segmental duplication and as a result three human-specific paralogs appeared, *NOTCH2NLA, NOTCH2NLB, NOTCH2NLC* and *NOTCH2NLR* (Fiddes et al., 2018; Suzuki et al., 2018). It was previously shown that *NOTCH2NL* is differentially expressed in neural stem and progenitor cells of fetal human neocortex and when this gene is expressed through electroporation in mouse embryos it promotes an increase in basal progenitors cell cycling (Florio et al., 2018). Furthermore, it has been shown that *NOTCH2NL* expression in mouse and human cortical organoids downregulates neuronal differentiation genes reducing differentiation of neural progenitors and increasing the final number of neurons (Fiddes et al., 2018). In addition, it was found that the presence of NOTCH2NL can block the expression of the Notch receptor *DLL1*, reducing neuronal differentiation (Suzuki et al., 2018). Altogether, these findings suggest that gene duplications have probably played an important role in the evolution of human-specific developmental mechanisms underlying cortical evolution. Altogether these studies support the adaptive role of duplications in human evolution (Iskow et al., 2012), since both non-coding (Kostka et al., 2010) and coding (Hahn et al., 2007; Han et al., 2009) sequences in duplicated loci show signatures of positive selection.

Besides large duplications, human-specific duplications and deletions of DNA shorter than one kilobase are widespread and encompass approximately 3.5% of the human genome (Britten, 2002; Chimpanzee Sequencing Analysis Consortium, 2005; Varki and Altheide, 2005). These rearrangements contribute more base pairs than do individual DNA substitutions to human-chimp differences, but fewer than larger chromosomal variants. It has been shown that indels can have critical functional effects, by modifying or completely deleting conserved developmental enhancers and rendering altered human phenotypes. For instance, a polymorphic 13 base pair insertion in a sonic hedgehog ZRS limb enhancer induced preaxial polydactyly and the appearance of triphalangeal thumbs (Laurell et al., 2012). A genome-wide analysis found 510 highly conserved sequences that were lost in the human lineage. Most of these lost sequences were non-coding, and included a sensory vibrissae and penile spine enhancer for the androgen receptor gene and a transcriptional enhancer active in the SVZ of the developing cortex located near the tumor suppressor gene *GADD45G* (McLean et al., 2011).

#### Point Changes in Coding and Non-coding Sequences

The human and chimpanzee genomes accumulated since the split of these two lineages more than 30 million single nucleotide substitutions corresponding to the 1.2% of the human genome. Approximately half of these substitutions arose on the human lineage and the majority of them correspond to non-coding DNA (Chimpanzee Sequencing Analysis Consortium, 2005).

##### Coding Changes

According to the evolutionary theory most substitutions are nearly neutral and therefore are unlikely to have contributed to the emergence of uniquely human traits. In order to identify the genetic bases underlying functional differences in humans, research focused initially on the identification of non-synonymous changes occurred in individual protein coding sequences that may lead to the appearance of novel protein functions or the origin of human-specific gene loss of function or pseudogenes. Comparison of nonsynonymous to synonymous substitution rates allows us to identify genes evolving under positive selection. Several studies focused on studying the evolution of genes in the human lineage identified brain expressed genes evolved that faster in humans (Dorus et al., 2004; Yu et al., 2006). However, the first comparative studies of humans and chimpanzees genomes also focused on protein-coding differences and found that positive selection in humans impacted mostly on genes involved in immunity, sensory perception, and reproduction but did not find a particular evolutionary trend in brain expressed genes in the human lineage (Clark et al., 2003; Bustamante et al., 2005; Nielsen et al., 2005). Other studies used population genetic data (Racimo et al., 2014) to identify genes that underwent positive selection after modern humans split from Neanderthals and Denisovans (Meyer et al., 2012; Prüfer et al., 2014). It has been hypothesized that several developmental genes that acquired human-specific coding changes could be responsible for the emergence of human-specific phenotypic traits (reviewed in Sikela, 2006; O'Bleness et al., 2012). These genes include the forkhead transcription factor *FOXP2*, which is associated with speech and language (Lai et al., 2001) and displays two human-specific amino acid substitutions that may have undergone positive selection (Enard et al., 2002; Zhang et al., 2002) although this consideration has been lately disputed (Ptak et al., 2009). In fact, more recent studies using human population data indicate that the pattern of variation in the *FOXP2* locus does not suggest a recent selective sweep affecting the acquired amino acids (Atkinson et al., 2018). To investigate the function of these two human-specific amino acids genetically modified mice carrying the two human-specific amino acids in the *FOXP2* were generated. These *FOXP2* humanized mice showed differences in cortico-basal ganglia circuits including dopamine levels, synaptic plasticity and dendrite morphology (Enard et al., 2009). The engineered mice also showed differences in ultrasonic vocalizations compared to wild type (Enard et al., 2009) but these differences do not persist in the adults (Hammerschmidt et al., 2015).

Another interesting example is *WDR62*, a gene that encodes a centrosome-associated protein expressed in neuronal precursors and in postmitotic neurons in the developing brain and whose absence cause microcephaly with simplified gyri and abnormal cortical architecture (Nicholas et al., 2010; Yu et al., 2010). *WDR62* shows accelerated evolution in the human terminal branch displaying six hominin-specific amino acids (Pervaiz and Abbasi, 2016). Although the functional consequences of these changes are yet to be understood, it is likely that the *WDR62* hominin-specific amino acids modified its function (Pervaiz and Abbasi, 2016).

##### Non-coding Evolution

At the time that more vertebrate genomes were sequenced, it became possible to implement models of DNA evolution to screen the entire human genome in the search for sequences that changed significantly (more than expected by chance) since divergence from chimpanzees (Pollard et al., 2006a,b; Prabhakar et al., 2006; Bird et al., 2007; Bush and Lahn, 2008). These studies mainly focused on the discovery of changes in non-coding regions that have a high probability to be functional. Thus, these investigations analyzed genomic regions that are highly conserved in non-human species (mammals or vertebrates) but changed significantly in humans. Before the appearance of epigenetic marks that help in the identification of non-coding functional elements (ENCODE Project Consortium et al., 2007; Kellis et al., 2014), using this signature of negative selection in other species helped to identify putative regulatory sequences with constrained function (Schwartz et al., 2000; Ovcharenko et al., 2004; Siepel, 2005; Prabhakar et al., 2006). These studies collectively identified over 2,500 non-coding regions defined as Human Accelerated Regions (HARs) (Capra et al., 2013; Hubisz and Pollard, 2014), most of which were likely shaped by positive selection although some of them show signatures of non-selective mechanisms such as GC-biased gene conversion (Pollard et al., 2006a; Katzman et al., 2010; Ratnakumar et al., 2010; Sumiyama and Saitou, 2011; Kostka et al., 2012). Furthermore, similar approaches have also been used to analyze regions of the human genome that changed significantly since divergence from archaic hominins (Green et al., 2010). It was found that HARs are enriched for substitutions that antecede the split from Neanderthals and Denisovans, suggesting that our genome did not evolve especially rapidly in the course of the emergence of modern humans (Burbano et al., 2012; Hubisz and Pollard, 2014). HARs have a distinctive genomic distribution since they cluster nearby regulatory genes including transcription factors expressed during development (Capra et al., 2013; Kamm et al., 2013b). These findings suggest that HAR mutations could potentially lead to the modification of developmental gene regulatory networks and thus, underlie the evolution of unique human traits. Interestingly, the gene that accumulates the largest number of HAR in the human genome is the neurodevelopmental transcription factor *NPAS3 (Neuronal PAS domain-containing protein 3)*, a gene that has been associated with several neurological diseases in humans (Pickard et al., 2005, 2009; Macintyre et al., 2010). In addition, *NPAS3* is expressed in telencephalic progenitor domains of the cortex, and the caudal and medial ganglionic eminences (CGE and MGE, respectively), and later in immature and mature cortical interneurons (Erbel-Sieler et al., 2004; Batista-Brito et al., 2008). In fact, it has been shown that *NPAS3* regulates neurogenesis in the brain and particularly that *NPAS3* mutants display reduced numbers of interneurons in the cortex (Stanco et al., 2014). Moreover, it has been shown that 11 out of the 14 HARs located in *NPAS3* introns, were capable of driving reproducible expression of a reporter gene in the CNS of transgenic zebrafish (Kamm et al., 2013b). Further studies showed that one of these regions (2xHAR.142) drove the reporter gene LacZ expression to an extended region of the developing anterior telencephalon in comparison with the chimpanzee and mouse ortholog sequences when tested in transgenic mice (Kamm et al., 2013a). This is a salient example among the currently small catalog of regulatory regions carrying human-specific changes that likely modified human-specific expression patterns of brain developmental genes.

More recently, it has been also shown that HARs accumulate in a topologically associated domain encompassing the gene *FOXP2* (Caporale et al., 2019). In fact, introns and intergenic regions of *FOXP2* harbor 12 HARs, several of which act as transcriptional enhancers in the nervous system in expression assays in transgenic zebrafish and mice. Moreover, two of these regions drove the reporter gene to *FOXP2* expressing cells in the developing brain and also display different expression patterns when compared with chimpanzee ortholog regions, indicating that the accelerated evolutionary process that they underwent in the human lineage are likely to have functional consequences (Caporale et al., 2019).

Boyd et al. (2015) have recently selected the HAR ANC516 previously identified (Bird et al., 2007) that they renamed as HARE5 for functional studies. This element located near the Wnt receptor *Frizzled 8* (*FZD8*) gene displays differential enhancer activity in the developing cortex of transgenic mice (Boyd et al., 2015) depending on whether HARE5 was from human or chimpanzee origin. In fact, the human sequence drives reporter gene expression in a more robust way and in an earlier developmental time point than the ortholog chimpanzee sequence in the developing cortex. Then, the authors generated transgenic mice carrying the chimp or the human HARE5 sequences controlling the expression of the mouse *Fzd8* coding sequence and analyzed comparatively several features of cortical development. Although this approach did not control for positional effects on the transgenics the results are worth to be mentioned. Overexpression of *Fzd8* controlled by human HARE5 produced a faster cell cycle in neuronal progenitors and led to increased neocortical size compared with mice where *Fzd8* is driven by chimpanzee HARE5 (Boyd et al., 2015). Although these results probably represent a step forward to understanding human brain evolution, further demonstration of how HARE5, NPAS3-HARs, or FOXP2-HARs impacted in human evolution still requires additional studies. An important issue to consider is that we still lack information about the expression pattern of *FZD8, FOXP2*, and *NPAS3* in human and chimpanzee developing brains. Thus, we do not know if these genes are in fact differentially expressed in these two species. In addition, it would be very informative to generate genetically engineered mice strains carrying human versions of HARE5 and other differentially expressed HARs replacing their mouse ortholog region to then analyze brain size, neuronal cell counts, and cognitive and behavioral traits.

A recent study integrated previously identified three-dimensional chromatin interaction map in developing human cortex (Won et al., 2019), which identified physical enhancer-promoter/gene interactions with HARs. This study identified the gene targets of HARs in the developing cortical plate of the human fetal cortex (Won et al., 2019). The authors found that the putative target genes of HARs are enriched in pathways involved in human brain development, dorsal-ventral patterning, cortical lamination, regionalization, and proliferation of neuronal progenitors which led them to suggest that multiple aspects of human brain development are subject to human-specific regulation (Won et al., 2019).

### Genetics of Human Cognitive Abilities

Regarding the genetics underlying the evolution of human cognitive abilities, in the last years some advances have been made into the identification of genetic loci relative to human cognitive function. In fact, Davies et al., found 148 genetic loci associated with general cognitive function using data from different large datasets like the UK biobank, CHARGE and COGENT consortia (Davies et al., 2018). Another recent study analyzed the expansion of cognitive networks in the human brain and the expression in these networks of genes associated to HARs (Wei et al., 2019). These authors found that HAR-associated genes are differentially expressed in higher-order cognitive networks in humans compared to chimpanzees and macaques (Wei et al., 2019). There is no doubt that these works will help to identify important genes and pathways that have played an important role into the evolution of our salient cognitive capacities.

## Concluding Remarks and Future Directions

Through this journey across the history of our cortex we can conclude that several key steps were necessary to render the mammalian neocortex that in some lineages reached a high degree of development and where highly-elaborated cognitive capacities are a distinctive feature. First, the appearance of the six-layered neocortex that probably happened in an ancestor of all mammals before the split of monotremes approximately between 240 and 180 mya was a cornerstone in the evolution of the organization of the basic plan of the mammalian neocortex. In this plan, the SVZ plays a fundamental role in the development of this six-layered neocortex. Then, the split and specialization of the SVZ seems to be the developmental mechanism that allowed the appearance of species with a high degree of encephalization and gyrencephaly, although it seems that the ancestor of all mammals possessed a gyrencephalic brain. However, more comparative studies will be necessary to help us to complete the puzzle and to better understand the molecular and cellular mechanisms underlying the emergence of the mammalian brain first and then brains with salient cognitive capacities. We still know very little about the genetic differences that led to the appearance of mammals and to the evolution of the distinctive characters of this group, particularly its brain. In the last years the explosion of the genomic era and the availability of genome sequences of many species of mammals and other vertebrates has enabled genome-wide comparisons among species and to detect genetic changes that emerged across their evolution. However, we need to understand how these genomic changes translate into gene expression differences or protein function modifications. Thus, it is important to perform comparative functional studies among different species that will help us to understand the phenotypic consequences of these genetic changes.

In this regard, the recent incorporation of different reptile species as animal models is helping us to understand the particular characteristics of the reptile brain and perform comparative studies to mammals illuminating in this way key aspects of mammalian brain evolution (Nomura et al., 2013b). In this sense, the development in the last years of several technologies will help to disentangle the evolutionary history of the mammalian brain. For instance, the possibility of studying brain organoids instead of animal models that are somewhat complicated due to several reasons including difficulty in laboratory reproduction promise to be crucial into understanding better brain developmental mechanisms in several lineages (Lancaster and Knoblich, 2014; Qian et al., 2019). However, some aspects of the development of the cortex are difficult to model in brain organoids, thus, this technique has to be used with caution and should be combined with the use of *in vivo* models that allow to model development in a more real system (Marx, 2020). In this sense, recent improvements to the protocols used to culture brain organoids are making them more complex and dynamic incorporating aspects of development that better mimic *in vivo* conditions (Shou et al., 2020). Moreover, the recent implementation of brain organoids from different primates is allowing us to model human brain evolution in a dish and to better understand how genetic differences translate into gene expression and phenotypic differences (Pollen et al., 2019).

In addition, high-throughput sequencing techniques such as RNAseq are allowing to perform comparisons of transcriptional landscapes of different species and thus pinpoint some fundamental genetic pathways that were modified in the different lineages. Moreover, single-cell RNAseq gives the possibility of exploring the gene expression program of a given cell and then comparing particular kinds of cells across different species. These techniques promise in the near future to help us understand the different genetic pathways that are activated in different cells across species to render differences in brain development.

Moreover, the development of CRISPR/Cas technologies that allow to genetically engineer almost any organisms (Gilbert et al., 2014; Zheng et al., 2014) will be crucial to understand how lineage-specific genetic modifications can impact on molecular pathways to finally render anatomic and functional changes in the mammalian cortex.

Finally, all this development in technology will help us to advance in knowledge and to better understand an essential piece of mammalian evolution: the mammalian brain.

## Author Contributions

The author confirms being the sole contributor of this work and has approved it for publication.

## Conflict of Interest

The author declares that the research was conducted in the absence of any commercial or financial relationships that could be construed as a potential conflict of interest.
